# Coastal Hypoxia in the Indian Ocean: Unraveling Drivers of Spatio‐Temporal Variability

**DOI:** 10.1111/gcb.70378

**Published:** 2025-08-06

**Authors:** Fan Yang, Laure Resplandy, Yangyang Zhao, Sam Ditkovsky

**Affiliations:** ^1^ Department of Geosciences and High Meadows Environmental Institute Princeton University Princeton New Jersey USA; ^2^ Program in Atmospheric and Oceanic Sciences Princeton University Princeton New Jersey USA

**Keywords:** coastal hypoxia, hazard mapping, Indian Ocean, interannual hypoxia, intraseasonal hypoxia, oxygen variability, seasonal hypoxia

## Abstract

Coastal hypoxia in the densely populated Indian Ocean regions has dramatic consequences for ecosystems and ecosystem services such as fisheries and coastal protection. However, inadequate understanding of the spatio‐temporal variability of coastal hypoxia in the region, and its physical and biological drivers, poses a major challenge for anticipating these risks. Here we use *in‐situ* observations and a high‐resolution (1/12 degree) biophysical model of the Indian Ocean to map areas with heightened vulnerability to coastal hypoxia and identify the drivers of coastal oxygen dynamics. We find strong regional disparities in the mechanisms and temporal scales that govern coastal hypoxia: seasonal upwelling in the eastern Arabian Sea, interannual upwelling/downwelling linked to Indian Ocean Dipole events in the eastern Bay of Bengal, intraseasonal fluctuations introduced by coastal currents, Kelvin waves, and eddies along the western Bay of Bengal, and biologically driven oxygen variations at the mouth of major rivers in the northern Bay of Bengal (e.g., Ganges–Brahmaputra and Irrawaddy–Sittang Deltas). This basin‐scale mapping identifies regions where intraseasonal hypoxia (e.g., western Bay of Bengal, river deltas) makes prediction of these events challenging and calls for intense monitoring, and regions where seasonal and interannual hypoxia (e.g., eastern Arabian Sea, eastern Bay of Bengal) facilitates the prevention of the adverse impacts on coastal ecosystems and their services.

## Graphical abstract

We map coastal hypoxia across the northern Indian Ocean and reveal regional contrasts in their severity, drivers, and timescales at which they occur. This basin‐scale analysis highlights regions where hypoxia is triggered on short intraseasonal timescales—such as the western Bay of Bengal and major river deltas—challenging prediction and underscoring the need for enhanced monitoring. In contrast, regions where hypoxia is more predictable and returns seasonally every year or interannually during dipole years—such as the eastern Arabian Sea and eastern Bay of Bengal—present opportunities for early warning systems and targeted mitigation.

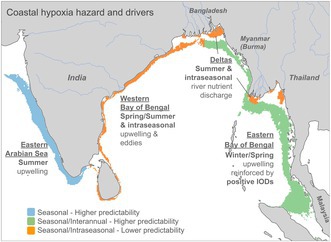

## 1 Introduction

Hypoxia is one of the most significant and urgent threats to coastal marine ecosystems (Vaquer‐Sunyer and Duarte 2008; Pörtner 2010; Rabalais et al. 2010). The occurrence of coastal hypoxia, commonly known as “dead zones,” has increased worldwide in recent decades (Gilbert et al. 2010; Conley et al. 2011; Altieri and Gedan 2015; Deutsch et al. 2024; Breitburg et al. 2018; Barth et al. 2024). Coastal hypoxic waters significantly influence the distribution and characteristics of both benthic and pelagic ecosystems by directly affecting the physiological processes and behavioral patterns of organisms within these communities, compressing viable habitats, and having a profound negative effect on biodiversity and fisheries in coastal regions (Vaquer‐Sunyer and Duarte 2011; Laffoley and Baxter 2019; Thambithurai et al. 2019; Deutsch et al. 2020). For example, hypoxic conditions have been shown to significantly impair the survival and performance of commercially important species (e.g., Tomasetti et al. 2023; Donelan et al. 2023), or reshape coral reef communities, leading to shifts toward more stress‐tolerant species, a loss of reef biodiversity and in extreme cases mass mortality (Lucey et al. 2024; Altieri et al. 2017). Furthermore, low oxygen levels promote denitrification and the generation of nitrous oxide, adding to the global climate burden (Naqvi et al. 2006; Ji et al. 2015, 2018; Frey et al. 2020).

**FIGURE 1:**
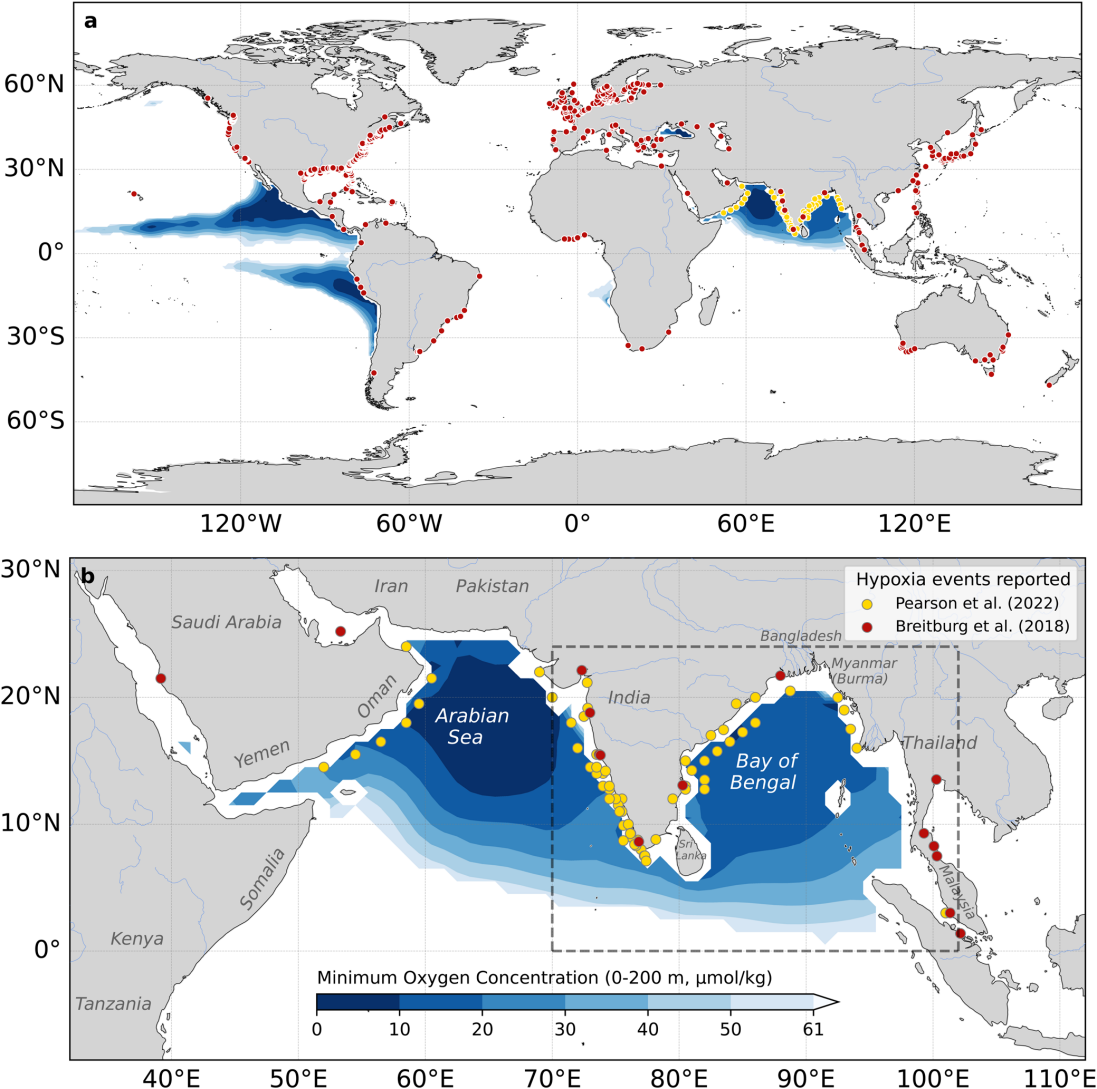
Distribution of minimum oxygen concentrations in the upper 200 m from Bianchi et al. (2012) in (a) global oceans and (b) the Indian Ocean, overlaid with coastal hypoxic sites (oxygen < 61 μmol/kg) reported by Breitburg et al. (2018) (red dots) and Pearson et al. (2022) (yellow dots). The dashed box in (b) indicates the analysis domain. Map lines delineate study areas and do not necessarily depict accepted national boundaries.

In recent decades, coastal hypoxia sites (hypoxia defined as oxygen levels lower than 61 μmol/kg) have been extensively documented, with over 500 reported sites globally since 1950 in the review by Breitburg et al. (2018). The reported sites in global syntheses are predominantly concentrated in coastal regions of the United States and Europe (78% of the total hypoxia sites, see red dots in Figure 1a). The number of hypoxic sites reported in the Indian Ocean is comparatively limited, with for instance Breitburg et al. (2018) reporting only 8 coastal hypoxic sites in the northern Indian Ocean (see red dots in Figure 1b). Yet, a more recent study, focusing on the Indian Ocean, identified 109 hypoxic sites published in previous literature (yellow dots in Figure 1b; Pearson et al. 2022), highlighting that coastal hypoxia in the northern Indian Ocean has been previously under‐reported and likely overlooked.

The Indian Ocean is particularly vulnerable to coastal hypoxia due to a combination of human‐induced and natural factors (Zhang et al. 2010; Laufkötter et al. 2017; Naqvi 2021). The region hosts the second‐largest oxygen‐minimum zones (OMZs) globally (Stramma et al. 2008, 2010; Resplandy et al. 2012; Rixen et al. 2020) and accommodates approximately one‐third of the world's population (Roy 2019; Zeller et al. 2023) potentially contributing to coastal eutrophication and increasing the occurrence of hypoxia (Rabalais et al. 2009, 2014). Furthermore, the Indian Ocean experiences significant climatic variability on various timescales, such as seasonal variation associated with the monsoons, interannual variation associated with the Indian Ocean Dipole (IOD), and short‐term processes such as cyclonic and anticyclonic eddies (Mukherjee et al. 2014; Phillips et al. 2021; Xu et al. 2019). Mesoscale eddies, in particular, have been shown to play a critical role in modulating OMZ dynamics in the Arabian Sea (Resplandy et al. 2012; Lachkar et al. 2016, 2018). Recent research based on *in*‐*situ* observations (Pearson et al. 2022) and a physical–biogeochemical regional model (Vallivattathillam et al. 2017) revealed that the IOD, a coupled ocean–atmosphere interannual phenomenon (Saji et al. 1999), can either amplify or suppress the seasonal coastal hypoxia hazard by modulating coastal seasonal upwelling/downwelling in the eastern Arabian Sea and the eastern Bay of Bengal. Yet, little is known about the factors controlling coastal hypoxia on shorter time scales and in regions where observations are sparse, such as the Bay of Bengal or major river deltas. For instance, episodic events are reported along the western Bay of Bengal and cannot be explained by seasonal dynamics (Pearson et al. 2022; Breitburg et al. 2018). Observations suggest that storm‐induced mixing, mesoscale eddies (Xu et al. 2019) and algal blooms, such as those caused by the dinoflagellate *Noctiluca scintillans* in the southwestern coast of the Bay of Bengal (Raj et al. 2020), influence coastal oxygen levels, potentially contributing to occasional hypoxia and stressing marine ecosystems. The scarcity of these observations limits, however, our understanding of the drivers controlling the occurrence and frequency of coastal hypoxic events, as well as our ability to anticipate and mitigate their impacts on ecosystem and ecosystem services.

Here, we use a high‐resolution biophysical model of the Indian Ocean (1/12 degree, Liao et al. 2025), combined with available *in‐situ* observations, to investigate coastal oxygen dynamics and identify drivers of oxygen variability and coastal hypoxia over timescales ranging from days to years. We show that the model reproduces the observed patterns of coastal hypoxia reported in the literature and quantify the spatial and temporal patterns of coastal hypoxia occurrence. We identify a strong contrast in the time‐scales as well as the physical and biological drivers of oxygen variability between the eastern Arabian Sea (EAS), western Bay of Bengal (WBoB), eastern Bay of Bengal (EBoB) and the major river deltas of the Bay of Bengal (BoB‐DR, Ganges–Brahmaputra and the Irrawaddy–Sittang deltas). Finally, we discuss the implications for the region's vulnerability to hypoxic events.

## 2 Methods

### 2.1 Regional Ocean Circulation Biogeochemical Model

We use the hindcast simulations from the regional ocean biogeochemical model of the Indian Ocean, MOM6‐COBALT‐IND12 (Liao et al. 2025). The model is based on the ocean‐ice Modular Ocean Model 6 (MOM6, Adcroft et al. 2019) and is coupled with the Carbon, Ocean Biogeochemistry and Lower Trophics biogeochemical module version 2 (COBALTv2, Stock et al. 2014, 2020). It features an intermediate complexity biogeochemical module, incorporating 33 tracers, including nutrients, phytoplankton groups, zooplankton groups, dissolved organic carbon pools, a specific detritus pool, and parameters such as oxygen and the carbonate system. The model also implicitly includes higher trophic level predators with their biomass dynamically linked to zooplankton prey abundance (Stock et al. 2020).

MOM6‐COBALT‐IND12 features a horizontal resolution of 1/12 degree over the northern Indian Ocean (32° E to 114° E and 8.6° S to 30.3° N), and can be considered “eddy‐resolving” in most of the domain although we note that some coastal regions such as the delta of the Narmada and Tapti rivers are probably under‐resolved (Hallberg 2013). The model was run for the period 1980–2020 after a 32‐year spin‐up (with repeat forcing looping over 1980–1987 years), using external forcing from the European Centre for Medium‐Range Weather Forecasts Reanalysis 5th Generation (ERA5) and the Global Flood Awareness System (GloFAS) reanalysis version 4.0 (Harrigan et al. 2020) for the freshwater discharge from rivers. The riverine fluxes of dissolved and particulate nutrients are constant annual mean values derived from Mayorga et al. (2010). A detailed model description including the observation‐based adjustments made to the river discharge is available in Liao et al. (2025). In this study, we investigate the 41‐year weekly model outputs to examine oxygen dynamics and coastal hypoxia. While the full simulation is used to assess long‐term hypoxia risk and variability, the most recent decade (2010–2020) is used to present detailed time series and budget analyses in the case study regions, as it allows clearer examination of variability from intraseasonal to interannual timescales. The model oxygen concentration evolves over time in response to physical transport, biological source and sinks and the air–sea oxygen exchange flux (*F*) following:

(1) $$\partial_{t}O_{2}={\left(\partial_{t}O_{2}\right)}_{\mathrm{phy}}+{\left(\partial_{t}O_{2}\right)}_{\mathrm{bio}}+F$$

The physical and biological contributions are defined as:

(2) $${\left(\partial_{t}O_{2}\right)}_{\mathrm{phy}}=-\nabla \cdot \left(uO_{2}\right)+\nabla \cdot \left(K\nabla O_{2}\right)$$

(3) $${\left(\partial_{t}O_{2}\right)}_{\mathrm{bio}}=\mathrm{WCR}+\text{Nitrif}+\mathrm{PP}+\mathrm{SOD}$$

where u is the three‐dimensional velocity; K is the three‐dimensional diffusivity tensor. PP is oxygen produced from primary production; WCR stands for water column respiration (including remineralization, zooplankton and high predator respiration); SOD stands for sedimentary oxygen demand (see Stock et al. (2020) for the sediment remineralization scheme used in COBALTv2); Nitrif is oxygen consumption due to nitrification (oxidation of ammonium).

### 2.2 Definition of Coastal Indian Ocean Regions

**FIGURE 2:**
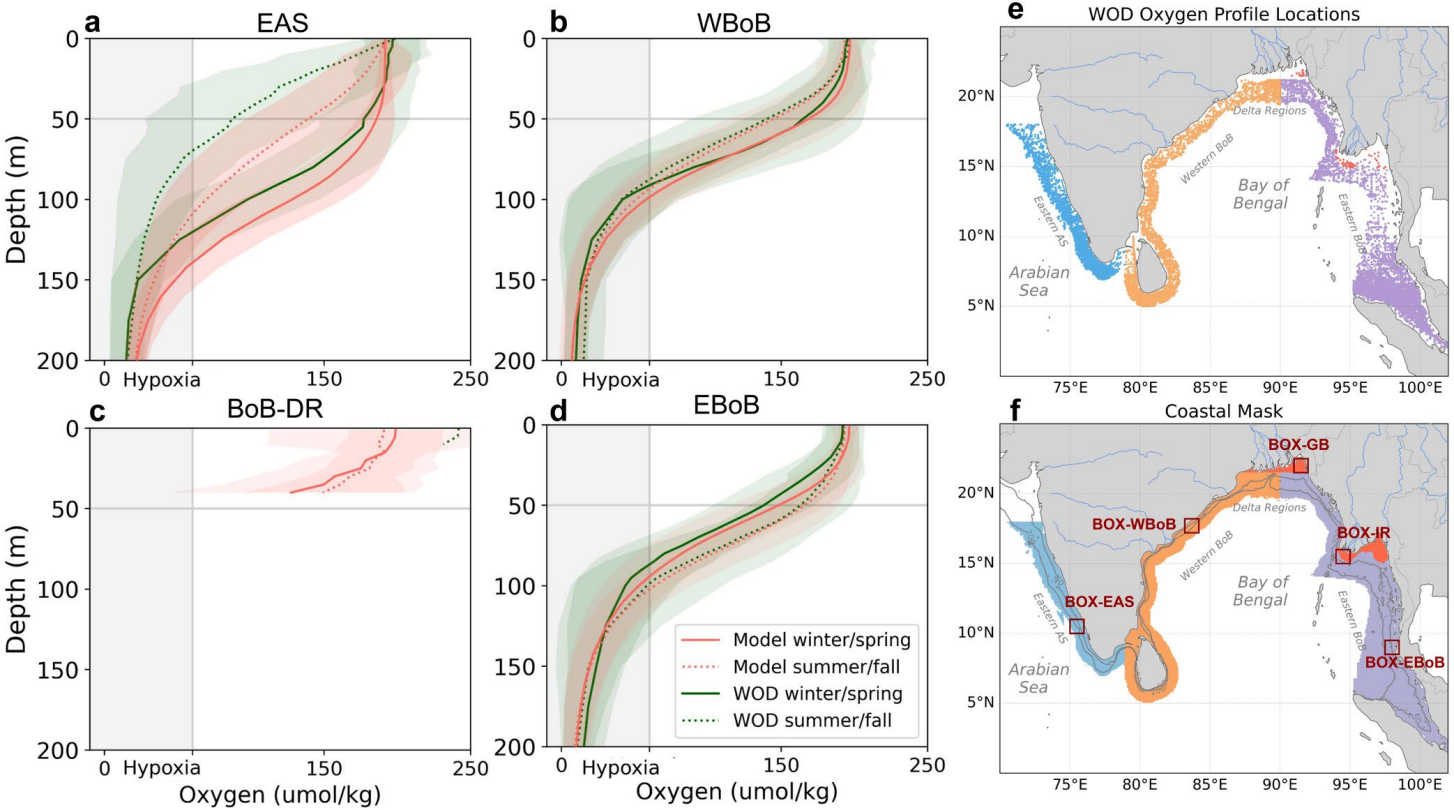
Comparison of modeled and observed oxygen profiles. (a–d) Oxygen profiles in observations (WOD, green) and model (MOM6‐COBALT‐IND12, red) averaged for winter/spring (solid) and summer/fall (dotted) seasons in each region over 1980–2020, with shading indicating ±1 spatio‐temporal standard deviation and gray shading indicating the hypoxia (O_2_ < 61 μmol/kg). (e) Oxygen profile locations in WOD in four coastal regions: Eastern Arabian Sea (EAS, blue), Western Bay of Bengal (WBoB, orange), northern Bay of Bengal Delta Regions (BoB‐DR, red) and Eastern Bay of Bengal (EBoB, purple). (f) coastal masks used for model analysis with grey lines indicating the 50 and 200 m isobaths and darkred boxes indicating the case study regions. Map lines delineate study areas and do not necessarily depict accepted national boundaries.

We define coastal regions using a distance of 100 km from the shore or the 1000 m isobath, selecting whichever is farther from the coast (Laruelle et al. 2017). In most coastal regions, the 1000 m isobath is located farther than 100 km from the coast, except in the western Bay of Bengal (Figure 2f). Our study concentrates on four major coastal regions: the Eastern Arabian Sea (EAS, blue), the Western Bay of Bengal (WBoB, orange), the BoB Delta Regions (BoB‐DR, red), and the Eastern Bay of Bengal (EBoB, purple) (see colors in Figure 2f). The EAS encompasses areas along India's western coast, situated south of 18° N and west of 79° E. The WBoB covers the coast of Sri Lanka and the eastern coast of India. The BoB‐DR region encompasses the region around the Ganges–Brahmaputra Delta and the Irrawaddy–Sittang Deltas (between 85° E and 97° E and bounded by an annual mean salinity limit of 29). Finally, the EBoB spans the coasts of Bangladesh, Burma, Thailand, and Malaysia.

We also use case study regions of 1° × 1° in each of the four key hypoxia‐affected areas. These regions include Box‐EAS in the eastern Arabian Sea, located in the south of India's western coast (10°–11° N, 75°–76° E); Box‐WBoB along India's eastern coast near Odisha (17.2°–18.2° N, 83.2°–84.2° E); Box‐EBoB on the southeast coast of the Andaman Sea (8.5°–9.5° N, 97.5°–98.5° E); and Box‐GB, located in the Ganges–Brahmaputra outflow region in the northern Bay of Bengal (21.5°–22.5° N, 91°–92° E), characterized by shallow coastal waters and significant riverine influence from the Ganges and Brahmaputra rivers. These regions were selected for their distinct hypoxia patterns, as indicated by the red boxes in Figure 2f, representing a range of frequency of hypoxia occurrence, from episodic to persistent.

### 2.3 Indian Ocean Dipole Phases

The IOD is characterized by warm anomalies in the western Indian Ocean and cool anomalies in the east during positive phases, and the opposite pattern during negative phases (Saji et al. 1999). We identify IOD phases using the Dipole Mode Index (DMI), following the definition by Pearson et al. (2022). These phases—positive, negative, and neutral—are defined to start on June 1 of a given year and end on May 31 of the following year, reflecting the typical fall peak of IOD activity. For simplicity, we refer to these periods using a shorthand notation, such as 1982/1983 to represent the period from June 1982 to May 1983. Positive IOD phases include 1982/1983, 1994/1995, 1997/1998, 2006/2007, 2012/2013, 2015/2016, 2018/2019, and 2019/2020, while negative IOD phases include 1992/1993, 1996/1997, 1998/1999, 2010/2011, and 2016/2017.

### 2.4 Characterization of Coastal Hypoxia and Oxygen Variability

We establish metrics to characterize hypoxic events, oxygen variability, and the mechanisms controlling these variations along the coastal regions of the Indian Ocean.

#### 2.4.1 Hypoxia Occurrence

We quantify the occurrence of hypoxia during the simulation period from 1980 to 2020 as the percentage of time when the minimum oxygen concentration in the upper 200 m falls below the hypoxic threshold (< 61 μmol/kg).

Areas vulnerable to coastal hypoxia are defined by hypoxia occurrence between 0.01% and 99% of the time. Specifically, we distinguish “Episodic” (occurrence between 0.01% and 10%), “Seasonal” (between 10% and 50%), and “Chronic” (between 50% and 99%). Furthermore, we consider regions where hypoxia is “Persistent” (more than 99% of the time) or where there is “No hypoxia” (< 0.01% of the time), which are not as vulnerable due to the systematic presence or absence of hypoxic conditions. This classification is consistent with the classification provided in observation‐based reviews of coastal hypoxia (e.g., Breitburg et al. 2018; Pearson et al. 2022) which also categorized sites into “Persistent” nearly always experiencing hypoxia; “Chronic” where hypoxia persists for at least an entire year; “Seasonal” where hypoxia recurs during one or two specific seasons annually; and “Episodic” where hypoxia events occur less frequently, typically lasting for less than 1 month per year. Additionally, these databases report sites labeled as “Unknown” for which the frequency of hypoxia events was not determined in the original publication.

#### 2.4.2 Oxygen Minimum Temporal Variability

We quantify the variability in oxygen minimum concentration in the upper 200 m in coastal waters over three timescales: seasonal using a 4‐ to 12‐month bandpass filter, intraseasonal using a 14‐ to 120‐day bandpass filter, and interannual using a 1‐ to 5‐year bandpass filter. Variability is quantified as two standard deviations (2σ) of the minimum oxygen concentration in the upper 200 m for the period 1980–2020. Timescales of oxygen minimum variability are useful to examine the ocean biological and/or physical processes at play and provide a more mechanistic approach than the classification used to report events in the literature (i.e., episodic, seasonal, chronic, and permanent). The two are, however, closely related with chronic events often aligning with interannual variations, while episodic events can occur on both intraseasonal and interannual timescales. Yet, as we show in our analysis, most regions are characterized by a superposition of processes with various timescales. For instance, oxygen minimum variability in river plume can be episodic/intraseasonal but always occur at the same time of year during peak river discharge.

#### 2.4.3 Timing of Highest Hazard

We calculate the percentage of time within each month from 1980 to 2020 that hypoxia (O_2_ < 61 μmol/kg) occurred in the upper 200 m. We then identify the month with the highest percentage of hypoxia, marking it as the month of highest hazard. We calculate the percentage of time hypoxic conditions occurred for each IOD phase (positive, neutral, and negative). A 4% threshold was applied to determine the phase with the highest hypoxia risk: if the occurrence in one phase exceeded the others by at least 4%, it was designated as the phase of greatest risk; otherwise, differences below 4% were classified as weak IOD influence. Given that we use weekly model output, a 4% difference translates to at least two additional data points indicating hypoxic conditions within a particular phase. This threshold was chosen to ensure that any identified phase‐specific influence on hypoxia risk is significant enough to be distinguished from typical variability and is not due to minor fluctuations. This approach allows us to map the month and the IOD phase during which the region experiences the most significant hazard of hypoxia.

#### 2.4.4 Oxygen Dynamics and Attribution to Physical and Biological Processes

We use the model oxygen budget (Equations (1), (2), (3)) to establish if physical or biological processes control changes in coastal oxygen. Budget over the depth range of 40–200 m for Box‐EAS, Box‐WBoB, and Box‐EBoB, and 5–20 m for the shallow, river‐influenced regions Box‐GB and Box‐IR, is computed to represent subsurface oxygen dynamics. We attribute the changes in oxygen using the sign of the total oxygen tendency ($\partial_{t}O_{2}$) and physical (${\left(\partial_{t}O_{2}\right)}_{\mathrm{phy}}$) and biological (${\left(\partial_{t}O_{2}\right)}_{\mathrm{bio}}$) contributions. Both biological activity and physical transport play a key role in controlling oxygen. In most regions, biology and physics act in opposite directions and largely compensate. Only in a few cases including in river plumes, physics and biology act together to reduce oxygen concentration. Specifically, we consider the anomalies of these contributions, calculated as the difference between the physical and biological tendency and their respective annual means. We distinguish three cases: changes are physically‐driven if the sign of $\partial_{t}O_{2}$ is the same as ${\left(\partial_{t}O_{2}\right)}_{\mathrm{phy}}$ but different from ${\left(\partial_{t}O_{2}\right)}_{\mathrm{bio}}$. Conversely, changes are biologically‐driven if $\partial_{t}O_{2}$ is the same as ${\left(\partial_{t}O_{2}\right)}_{\mathrm{bio}}$ but different from ${\left(\partial_{t}O_{2}\right)}_{\mathrm{phy}}$. Changes are controlled by both physical and biological changes when ${\left(\partial_{t}O_{2}\right)}_{\mathrm{bio}}$ and ${\left(\partial_{t}O_{2}\right)}_{\mathrm{phy}}$ have the same sign. This sign‐based attribution method identifies which process dominates when physical and biological drivers have opposing effects on oxygen. However, when both drivers act in the same direction, such as jointly contributing to oxygen decline, the method classifies both as actively contributing to the observed change.

#### 2.4.5 Detection of Mesoscale Eddies and Influence on Oxygen

We assess the role of mesoscale eddies on coastal oxygen levels by contrasting oxygen depth profiles in cyclonic and anticyclonic eddies. We employ the Pyeddy tracker with a 300 km cutoff wavelength (Mason et al. 2014) to identify mesoscale eddies and classified them as cyclonic or anticyclonic (see Figure S2). The eddy tracking algorithm uses sea level anomaly (SLA) to detect eddies through the identification of closed SLA contours (Mason et al. 2014). We then compute depth‐profiles of oxygen concentration and oxygen concentration anomaly (compared to the mean values) beneath the centroids of cyclonic eddies and anticyclonic eddies in the upper 200 m (Atkins et al. 2022).

To ensure consistent detection of hypoxic conditions across both shallow and deeper coastal regions, we quantify hypoxia occurrence, oxygen variability, and the timing of the highest hazard based on the minimum oxygen concentration in the upper 200 m. This approach avoids missing signals from shallow waters that would be excluded by depth‐averaged metrics and enables unified comparisons across regions. The depth ranges used for oxygen budget analyses in the case study regions are tailored to capture region‐specific subsurface layers where hypoxia is most likely to occur. Similar depth ranges have been used in previous studies to examine spatio‐temporal variability in coastal subsurface oxygen, such as Pearson et al. (2022), who used 50–200 m and found their results were not highly sensitive to changes in depth bounds (e.g., 40–200 m or 50–225 m).

### 2.5 Observations and Model Evaluation

A detailed evaluation of the model was performed in Liao et al. (2025), including the OMZs pattern which is well captured by the model (correlation coefficient between observation‐based and modeled oxygen concentrations between 300 and 700 m *r* = 0.94). We find that the model reproduces the basin‐scale distribution of hypoxic zones across the northern Indian Ocean, but tends to overestimate oxygen levels in the Arabian Sea, as seen in the simulated minimum oxygen concentrations in the upper 200 m (Figure S1).

We further evaluate oxygen in the coastal domain using observed oxygen profiles from the World Ocean Database version 2018 (WOD18; Boyer et al. 2018) and reported hypoxia sites compiled by Pearson et al. (2022) and Breitburg et al. (2018). We compare simulated oxygen profiles against observational data obtained from the WOD during winter/spring (December to May) and summer/fall (June to November) in the four study regions. The model captures the main seasonal patterns (lines) and the spatial–temporal variations (1 standard deviation in shading) observed in the four coastal regions (Figure 2a–d). Specifically, the model reproduces the winter/spring shoaling of the oxycline in the EBoB region (−15 m), as well as the summer/fall oxycline shoaling in the WBoB (−10 m) and EAS (about −40 m). We note, however, that the model tends to overestimate oxygen levels in the Arabian Sea, leading to a 30 m deeper oxycline compared to observations in the EAS coastal region (Figure 2a). This positive oxygen bias likely results from two main limitations: (1) low bias in primary productivity in this region, potentially yielding too low oxygen consumption (Liao et al. 2025) and (2) simplifications in the biogeochemical model COBALTv2, including the use of a uniform sinking speed for particulate matter, which prevents the model from capturing regional contrasts (e.g., fast‐sinking in Bay of Bengal vs. slow‐sinking in Arabian Sea) important for oxygen consumption (Al Azhar et al. 2017; Rixen et al. 2019). Consequently, the model may underrepresent hypoxic events in this region. In the BoB‐DR region near major river mouths, ocean depths are shallow, and < 50 m. Observational data in these areas is scarce, limiting direct comparisons (Figure 2c).

## 3 Results

### 3.1 Observed and Modeled Occurrence of Coastal Hypoxia

**FIGURE 3:**
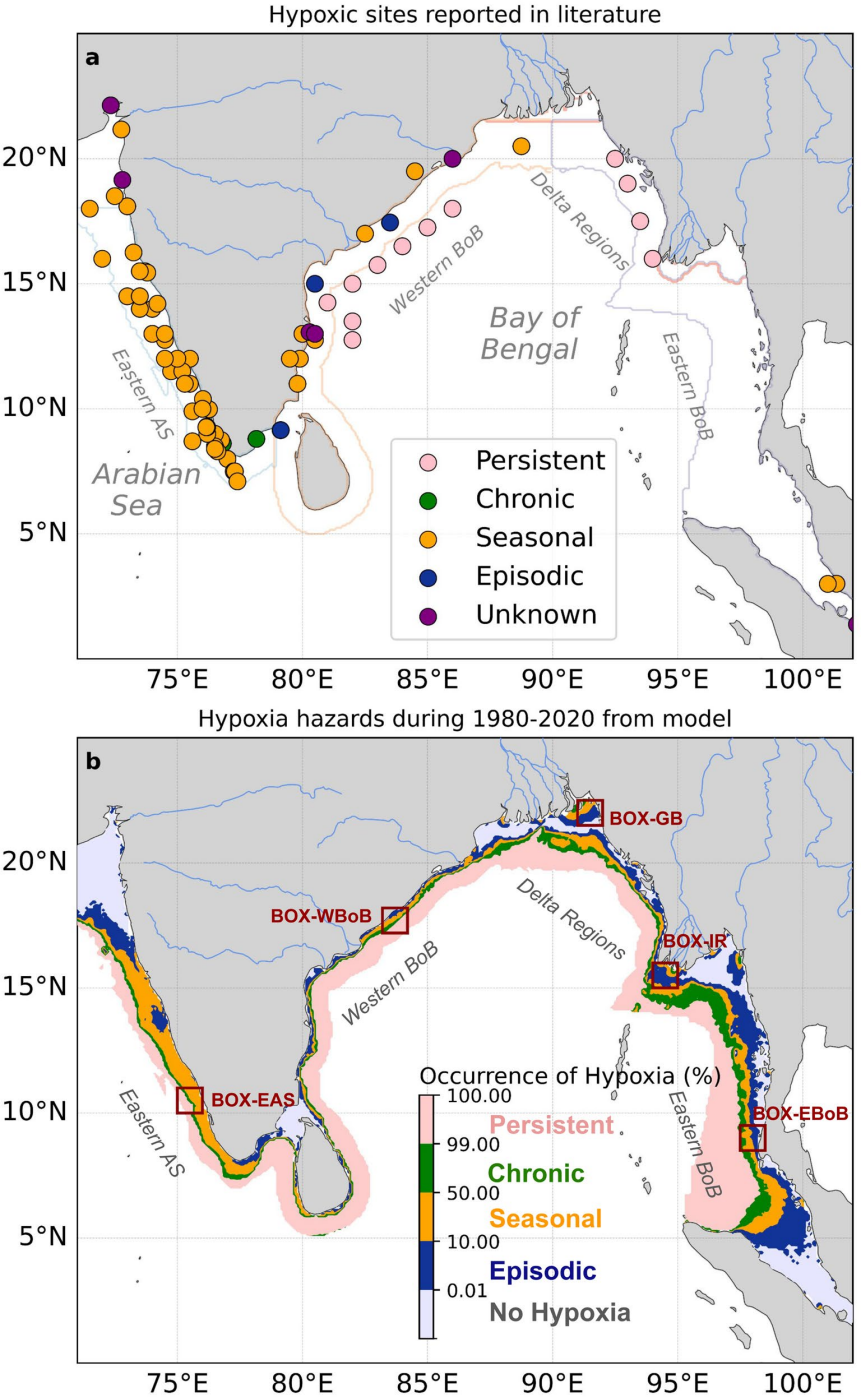
Hypoxia occurrence: (a) hypoxic sites from Pearson et al. (2022) with their reported occurrence: “Persistent” (nearly always present), “Chronic” (at least an entire year), “Seasonal” (in one or two seasons), “Episodic” (< 1 month per year), and “Unknown” when not identified. (b) hypoxia occurrence in the model (percentage of time during 1980–2020 period with minimum oxygen in upper 200 m < 61 μmol/kg) classified as “Persistent” (> 99%), “Chronic” (50%–99%), “Seasonal” (10%–50%), “Episodic” (0.01%–10%) and “No Hypoxia” (< 0.01%) for comparison to observations. Lines in panel a delimit the four regions: EAS, WBoB, BoB‐DR and EBoB (see details in Methods Section 2.2). Map lines delineate study areas and do not necessarily depict accepted national boundaries.

The northern Indian Ocean is vulnerable to coastal hypoxia, with a strong regional contrast in the temporal frequency of these events. Figure 3 illustrates both reported hypoxia sites in the literature (Pearson et al. 2022) and modeled occurrences of hypoxia within the studied regions, providing a comprehensive view of how hypoxic conditions manifest spatially and temporally. The reported hypoxia sites follow a regional pattern characterized by *seasonal* hypoxia in the eastern Arabian Sea (EAS), a mix of *seasonal* and *episodic* sites in the nearshore western Bay of Bengal (WBoB) and *persistent* hypoxia observed further offshore (Figure 3a). In contrast, the major deltaic regions of the Bay of Bengal (BoB‐DR) and the eastern Bay of Bengal (EBoB) have very few reports of hypoxia, with the exception of several *persistent* hypoxic sites identified between 15° N and 20° N in the EBoB. The low number of sites identified in BoB‐DR and EBoB regions is likely due to the lack of reporting.

The model reproduces the strong contrast between the EAS where *seasonal* hypoxia hazard is predominant and the WBoB where hypoxic occurrence follows a pattern increasing from *episodic* nearshore to *seasonal*, *chronic* and finally *persistent* further offshore, in agreement with published reports (Figure 3b). The model suggests that this same sequence is found in the WBoB and EBoB regions, with *episodic* to *chronic* conditions found on the shelf (isobath < 200 m) and *persistent* conditions in deeper waters (Figure 3b). River plume regions in BoB‐DR regions are characterized by a reverse sequence going from *chronic* at the river mouths to *seasonal* and *episodic* occurrence in the plume (Figure 3b). This indicates that river inputs control hypoxic conditions in these large river estuaries and adjacent shelf regions (Figure 3b). This spatio‐temporal mapping highlights the heterogeneity in hypoxia occurrence, indicative of heterogeneity in the drivers controlling hypoxia in the northern Indian Ocean.

### 3.2 Spatio‐Temporal Variability in Coastal Oxygen and Hypoxia

**FIGURE 4:**
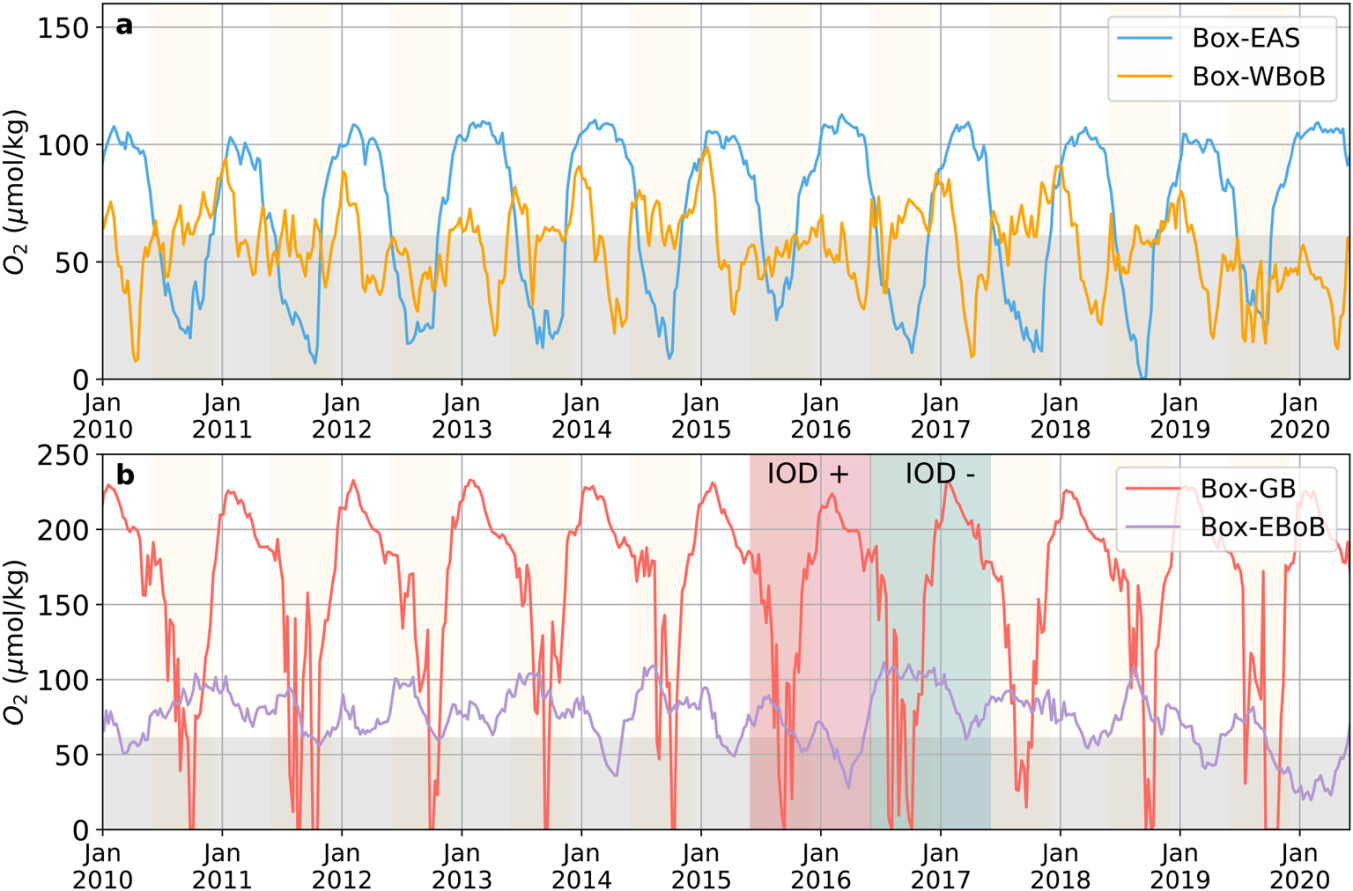
Time series of minimum oxygen concentrations in the upper 200 m in the four case studies regions (darkred boxes in Figure 2f): (a) Eastern Arabian Sea (Box‐EAS) and Western Bay of Bengal (Box‐WBoB), and (b) Delta Regions, specifically the Ganges–Brahmaputra river plume (Box‐GB) and Eastern Bay of Bengal (Box‐EBoB). The grey shading indicates hypoxic conditions (< 61 μmol/kg). Red and green shadings indicate the 2015/2016 positive and 2016/2017 negative IOD years.

**FIGURE 5:**
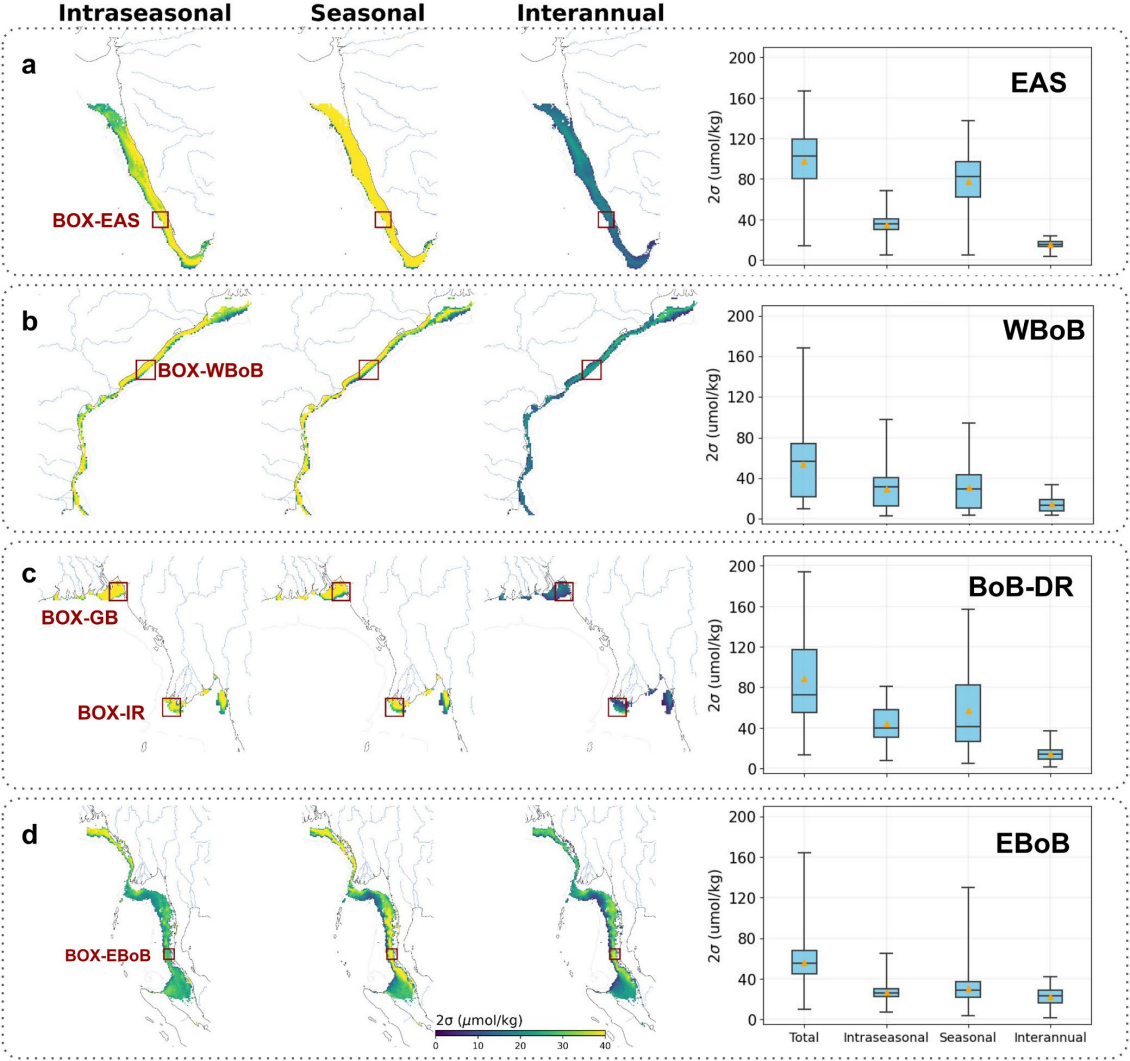
Oxygen minimum spatio‐temporal variability on intraseasonal (14–120 days), seasonal (4–12 months), and interannual (1–5 years) time‐scales in the (a) EAS, (b) WBoB, (c) BoB‐DR and (d) EBoB. Maps show the variability for each timescale. Boxplots show total, intraseasonal, seasonal and interannual variability averaged in the four regions (boxplot indicates first and third quartiles, median as line, mean as yellow dot). Variability is quantified by the two standard deviation (2σ) of oxygen minimum in upper 200 m filtered using bandpass filters over the 1980–2020 period (see Section 2). Only coastal regions vulnerable to hypoxia (0.01%–99% hazard in Figure 3b) are shown. Map lines delineate study areas and do not necessarily depict accepted national boundaries.

We illustrate the contrasts in the temporal occurrence of hypoxia (e.g., *episodic*, *seasonal*, *chronic*) across regions using four case studies located in the EAS, WBoB, BoB‐DR, and EBoB (see red boxes in Figure 3b for locations). In the EAS case study (Box‐EAS), oxygen concentrations show pronounced seasonal fluctuations, reaching hypoxic conditions every summer/fall (blue line in Figure 4a), consistent with the numerous reports of *seasonal* hypoxic events (Figure 3a). This seasonal hypoxia typically begins in June and persists through September, before oxygen levels recover during the winter months. In contrast, oxygen levels in the WBoB case study (Box‐WBoB) are characterized by both seasonal and intraseasonal variations (orange line in Figure 4a), consistent with reports of both *episodic* and *seasonal* hypoxic events in this region (Figure 3a). Seasonal hypoxia in Box‐WBoB peaks in spring, between March and May, while frequent intraseasonal fluctuations can drive abrupt oxygen declines within 2–4 weeks, highlighting the episodic nature of hypoxia in this region (Figures 5b and 7).

The case study in the Ganges–Brahmaputra plume region (Box‐GB) is characterized by systematic 2–4 weeks hypoxic conditions between July and October (Figures 5c and 9), consistent with the *episodic* and *seasonal* hypoxic occurrence simulated in river plumes (Figure 3b). Finally, the case study in the EBoB (Box‐EBoB) is located in a region of a mix of *chronic*, *seasonal* and *episodic* hypoxia characterized by relative low oxygen levels throughout the year, but also with significant interannual variability (purple line in Figure 4b). For example, during the 2015/2016 positive IOD event, hypoxic conditions were intensified, with oxygen levels dropping below 30 μmol/kg. In contrast, during the negative IOD years of 2016/2017, oxygen levels remained consistently above the hypoxia threshold and peaks of up to 110 μmol/kg persisting for an extended duration. This suggests that interannual climate variability, particularly the IOD, plays a crucial role in modulating the intensity of hypoxia in the EBoB region.

We generalize the results of these four case studies by quantifying the variability in coastal oxygen minimum concentration on seasonal (4‐ to 12‐month bandpass filter), intraseasonal (14‐ to 120‐day bandpass filter) and interannual (1‐ to 5‐year bandpass filter) timescales (Figure 5). We focus the analysis on coastal zones vulnerable to hypoxia (i.e., occurrence of hypoxia > 0.01% and < 99%, Figure 3b), and quantify variability using two standard deviations (2σ) of oxygen minimum concentration time‐series filtered for each time‐scale over the 1980–2020 period (see details in Section 2). The EAS and BoB‐DR show the highest variability in oxygen minimum concentrations, with 2σ ranging between 60 and 120 μmol/kg (interquartile ranges) in more than 50% of the area and variability that can reach up to 160–200 μmol/kg (Figure 5a,c). The WBoB and EBoB are characterized by milder variability in oxygen minimum concentrations, with 2σ ranging between 20 and 80 μmol/kg in approximately 50% of their area and maximum variability of about 160 μmol/kg (Figure 5b,d).

The timescales contributing to the total variability in oxygen minimum concentrations varies across the four regions. In the EAS, seasonal variability (median variability of ~80 μmol/kg) is the main driver of the high oxygen temporal variability, while intraseasonal (median of ~40 μmol/kg) and interannual (median of ~15 μmol/kg) variability contribute significantly less (Figure 5a). In the BoB‐DR and the WBoB, seasonal and intraseasonal contribute significantly and similarly (median variability of ~30 μmol/kg) to the total variability compared to the weaker interannual variability (median < 20 μmol/kg, Figure 5b,c). Finally, in the EBoB, all timescales contributes equally (median variability of ~30 μmol/kg), giving interannual variability a larger relative contribution than in the other regions (Figure 5d). The model suggests that the magnitude of oxygen minimum temporal variability is relatively homogeneous in space in the EAS and WBoB, but presents hotspots at the river mouths in the BoB‐DR and along the north–south coastlines in the EBoB (Figure 5).

### 3.3 Mechanisms Controlling Coastal Hypoxia

**FIGURE 6:**
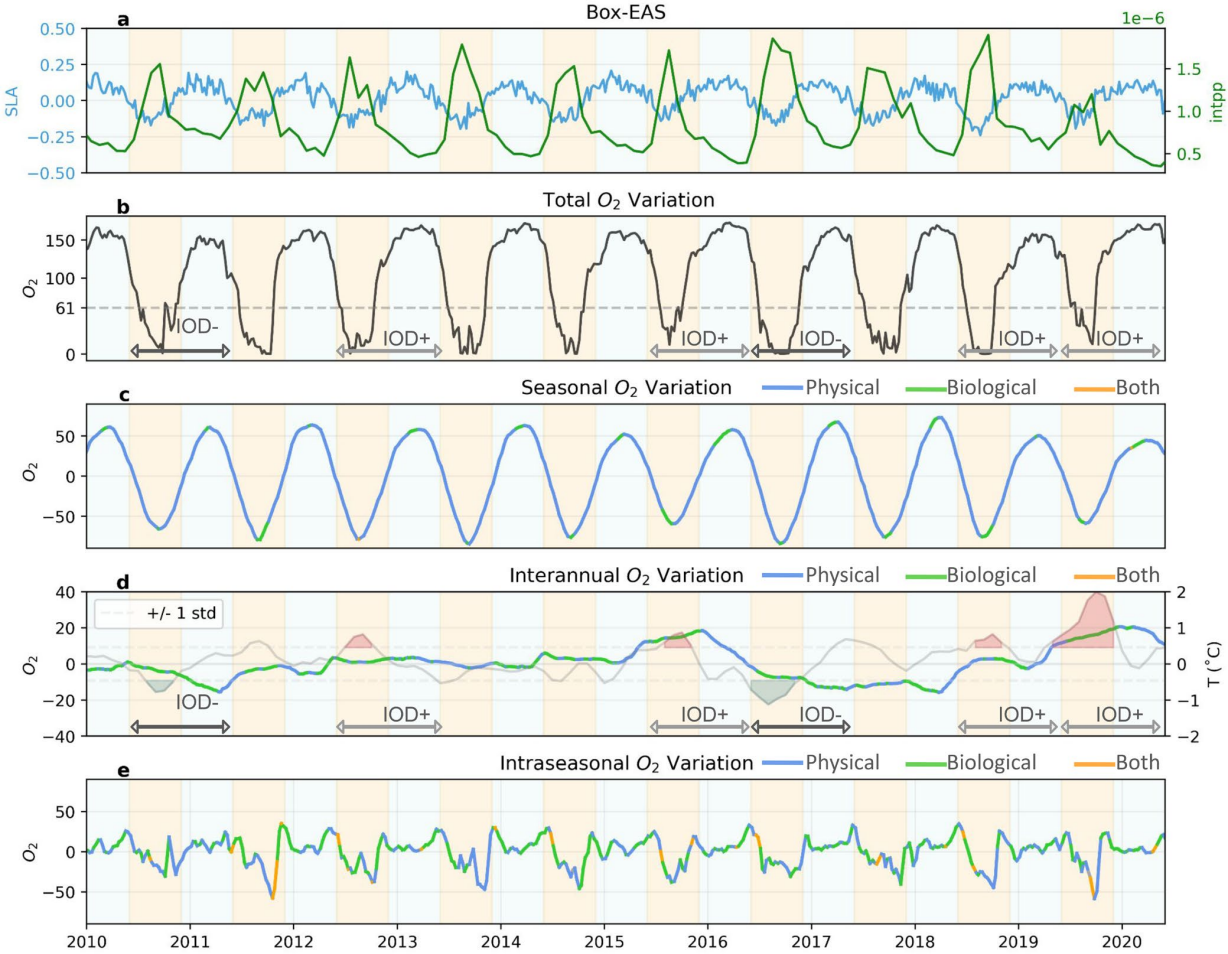
Oxygen dynamics in the eastern Arabian Sea case study region Box‐EAS from 2010 to 2020. (a) sea level anomalies (SLA, m) and integrated primary production in the upper 100 m (intPP, mol m^−2^ s^−1^); (b–e) Decomposition of oxygen variability (averaged between 40 and 200 m, μmol/kg) across total, seasonal, interannual, and intraseasonal timescales. The main drivers of oxygen variability on seasonal, interannual, and intraseasonal timescales are indicated by color: Blue where physical processes dominate, green where biological processes dominate, and orange where both physical and biological processes actively contribute to the oxygen changes. The yellow shading indicates the summer/fall period (June to November). In panel d, green and red shadings represent negative and positive IOD phases, respectively.

**FIGURE 7:**
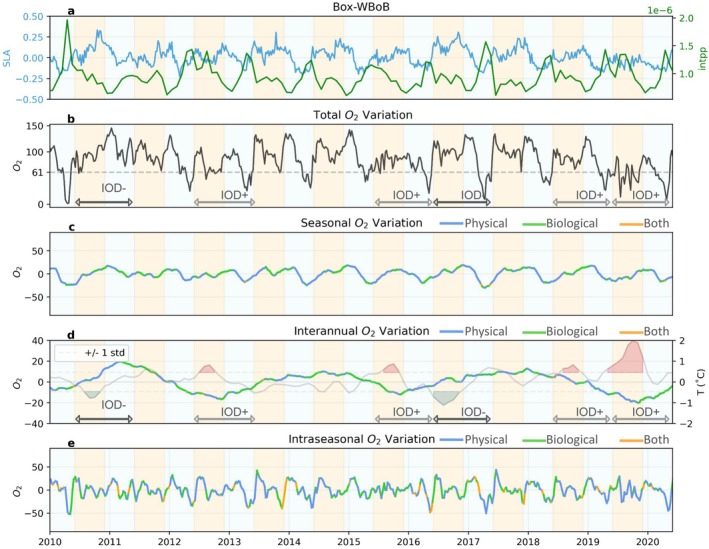
Same as Figure 6, but for the western Bay of Bengal case study region Box‐WBoB.

**FIGURE 8:**
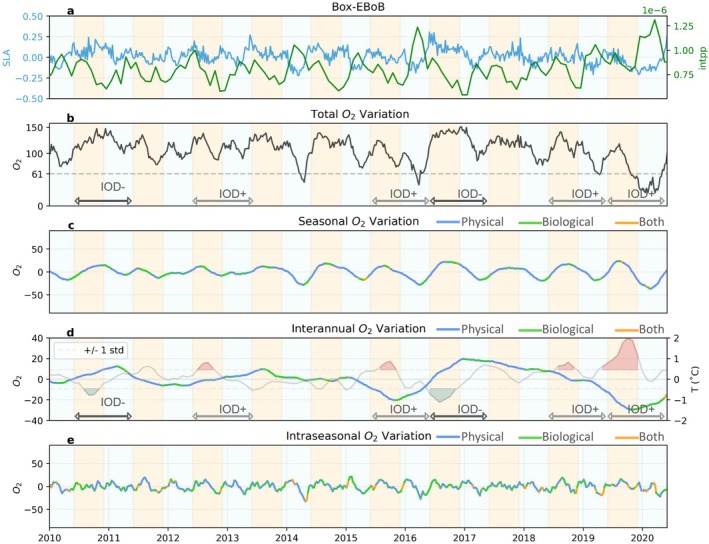
Same as Figure 6, but for the eastern Bay of Bengal case study region Box‐EBoB.

**FIGURE 9:**
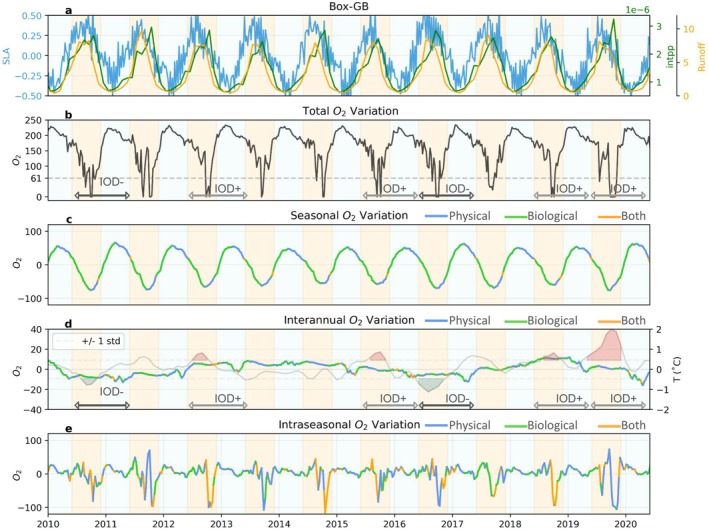
Same as Figure 6, but for the northern Bay of Bengal river delta regions Box‐GB, with oxygen variations averaged between 5 and 20 m depth.

We attribute oxygen variations on seasonal, interannual, and intraseasonal time‐scales to either physical or biological processes using the modeled oxygen budget in the four case study regions (see Section 2 for details). We first focus on the three continental shelf regions under the strong influence of coastal Kelvin wave dynamics (Box‐EAS, Box‐WBoB, and Box‐EBoB, Figures 6, 7, 8) before presenting the mechanisms in the Ganges–Brahmaputra delta region (Box‐GB, Figure 9). In all regions, both biological processes and physical transports are crucial in regulating oxygen dynamics (Figures S8–S11). Biological and physical processes generally act in opposite directions and partially compensate for each other's impact on oxygen levels, except during short periods of time or in river‐influenced regions, where they more often act together to reduce oxygen concentration. Here, we assess if physical transport, biological contribution, or both of them are the leading factor in controlling the seasonal, interannual, and intraseasonal decline in oxygen (see Section 2). We first present the drivers of variability at the seasonal timescale, which dominates the total variability in oxygen (Section 3.3.1). We then investigate how this seasonality is modulated over interannual timescales, primarily by the IOD (Section 3.3.2). Finally, we investigate intraseasonal sources of variability, with a focus on the region where intraseasonal drivers represent significant contributions to the total variability (Section 3.3.3).

#### 3.3.1 Upwelling Controls Seasonal Hypoxia on Continental Shelves

Seasonal hypoxia is a dominant feature across the northern Indian Ocean coastal waters (Figure 5). In the Box‐EAS region, the seasonal signal is particularly strong, with total oxygen concentrations decreasing dramatically from around 150 μmol/kg to near anoxic conditions every summer/fall (Figure 6b,c). This significant decline in oxygen levels corresponds to the timing of upwelling events, as indicated by negative SLA and the peak of primary production integrated over the upper 100 m (intPP) during summer/fall (Figure 6a). The model suggests, however, that the upwelling of low oxygen waters associated with the seasonal propagation of coastal Kelvin waves (Pearson et al. 2022) is the main driver of this decline (blue lines, indicating physical‐driven oxygen variations in Figure 6c and Figure S8). In the model, the upwelling corresponds to the combined effects of horizontal and vertical advection over the shallow shelf. The biological oxygen draw down expected in response to the bloom (respiration of organic matter in subsurface) plays a secondary role. In Box‐WBoB and Box‐EBoB regions, the seasonal signal is weaker, with oxygen decreasing in winter/spring by 20–40 μmol/kg in the Box‐WBoB and 10–50 μmol/kg in the Box‐EBoB depending on the year (Figures 7 and 8c). Similarly to the Box‐EAS, these oxygen declines coincide with upwelling motions (negative SLA) and enhanced intPP, but are largely controlled by the physical upwelling of low oxygen waters (blue lines, indicating physical‐driven oxygen decline in Figures 7c and 8c). We also note that, in the Box‐EBoB region, oxygen removal is primarily driven by horizontal advection and biological processes (Figure S6). On the shallow continental shelf with a gentle slope, upwelling moves low‐oxygen water horizontally along the shelf rather than vertically, making horizontal transport the main contributor to hypoxia in this area.

#### 3.3.2 Upwelling and Biological Contributions to Interannual Hypoxia on Continental Shelves

Interannual variability associated with IOD phases modulates the seasonal occurrence of hypoxia in all regions, but the magnitude and phasing of the impact varies across regions. Positive IOD phases can lower oxygen levels by about 20–50 μmol/kg in Box‐EBoB and Box‐WBoB, whereas negative IOD phases can lower oxygen levels by 10–20 μmol/kg in Box‐EAS (Figures 6, 7, 8). In the case of the Box‐EBoB region, hypoxic events are almost exclusively associated with positive IOD years (Figure 8b,d), while in other regions IODs mostly modulate the intensity of hypoxia. In all regions, oxygen interannual variations are alternately controlled by physical and biological processes (Figures 6, 7, 8: panels d). The magnitude of these variations and the balance between physical and biological contributions vary across the different IOD events. Yet, IOD phases with lower oxygen concentrations are generally characterized by lower SLA values and higher intPP than IOD phases with higher oxygen concentrations (Figure 8a,d), suggesting that IOD‐driven modulations of coastal Kelvin waves upwelling and the associated biological response both contribute to the interannual modulations in coastal oxygen and coastal hypoxia.

#### 3.3.3 Ocean Circulation Controls Intraseasonal Hypoxia in the Western Bay of Bengal

Intraseasonal variability in oxygen levels is particularly pronounced in the Box‐WBoB region (Figures 5 and 7c,e). Intraseasonal drops in oxygen concentrations in this region can reach up to 50 μmol/kg in spring of most years. These episodic events are largely controlled by physical processes, although biological processes provide an additional smaller contribution (Figure 7e and Figure S9). Negative SLA values (Figure 7a) during these spring events are consistent with intraseasonal variations in coastal undercurrents and upwelling motions reported in the literature (Mukherjee et al. 2014), and also with the signature of mesoscale eddies encroaching in coastal waters. In the model, we find that cyclonic eddies elevate low‐oxygen layers toward shallower depths, while anticyclonic eddies have the opposite effect (Figure S2). We note that this effect of eddies on oxygen is particularly strong in the WBoB, while its impact is much smaller in other regions. In contrast, intraseasonal oxygen variability in Box‐EAS and Box‐EBoB is less dominant relative to seasonal variability. In Box‐EAS, intraseasonal fluctuations can amplify oxygen decline by about half the magnitude of the seasonal signal (Figure 6e), while in Box‐EBoB, the contribution reaches up to 30 μmol/kg (Figure 8e). However, in both regions, these intraseasonal variations remain secondary and do not exceed the seasonal amplitude. As a result, intraseasonal drivers play a more limited role in shaping hypoxia dynamics in Box‐EAS and Box‐EBoB compared to Box‐WBoB.

#### 3.3.4 River Discharge and Biological Activity Control Hypoxia in River Deltas

The Ganges–Brahmaputra region behaves significantly differently from the other case study regions. The seasonal decline of oxygen during the summer/fall, coinciding with peaks in river runoff, leads to a drop in oxygen concentrations by 60–90 μmol/kg during this period (Figure 9c). The seasonal oxygen variations are initially controlled by physical processes in the spring (blue segments of the line during spring in Figure 9c, indicating physical‐driven oxygen decline). However, as summer progresses, biological processes become the main driver of oxygen depletion (green segments of the line during summer in Figure 9c, indicating biological‐driven oxygen decline). Nutrient‐rich river runoff stimulates high intPP, which peaks about 1 month after the peak in runoff (Figure 9a). The biological consumption of oxygen, fueled by intPP, intensifies through the summer, driving oxygen levels toward near‐anoxic conditions by late summer (Figure 9c). Although vertical mixing plays a role in oxygen replenishment, it cannot compensate for the high rate of biological oxygen consumption during this period (Figure S7). Biological processes and horizontal advection are the primary factors removing oxygen during this period. On intraseasonal timescales, oxygen declines can be even more dramatic, with drops of more than 100 μmol/kg over approximately 2 weeks, reinforcing the seasonal oxygen decline (Figure 9e). Both physical and biological processes contribute to these intraseasonal variations, with the model simulating pulses in biological oxygen consumption but also in the horizontal transport of low oxygen waters (see Figure S7). On the interannual scale, while the signal in Box‐GB is weaker than in other regions, additional oxygen declines or increases of up to 10 μmol/kg can occur. We note that similar dynamics are at play in the Irrawaddy–Sittang Delta (Box‐IR), with even stronger contributions from biological processes during periods of peak river discharge (see Figure S3).

#### 3.3.5 Highest Hypoxia Hazard: Regions and Timing

**FIGURE 10:**
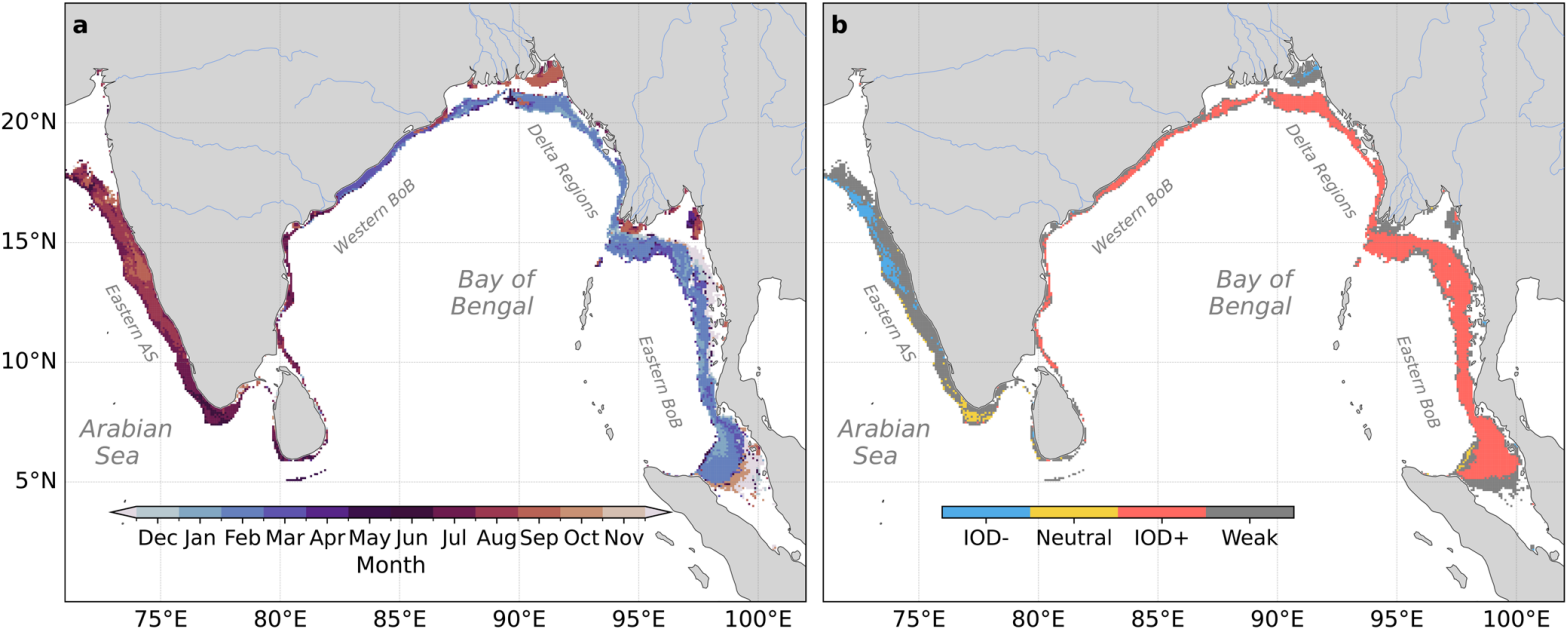
Timing of highest hypoxia hazard over 1980–2020: (a) months during which these events are most likely to occur, and (b) Indian Ocean Dipole (IOD) phase during which these events are most likely to occur, identified using a 4% threshold (i.e., the likelihood of hypoxia in a given phase is at least 4% higher than in other phases). Differences among phases below 4% are categorized as weak IOD influence. Positive IOD phases include the years 1982/1983, 1994/1995, 1997/1998, 2006/2007, 2012/2013, 2015/2016, 2018/2019, and 2019/2020 while negative IOD phases include the years 1992/1993, 1996/1997, 1998/1999, 2010/2011, and 2016/2017. Only coastal regions vulnerable to hypoxia events (occurrence likelihood between 0.01% and 99% in Figure 3b) are shown. Map lines delineate study areas and do not necessarily depict accepted national boundaries.

Figure 10 summarizes the basin‐scale patterns of hypoxia hazard by showing the spatial extent, the month, and IOD phase with the highest hypoxia hazard, all highlighting the regional contrasts in the processes driving hypoxia. Large river deltas of the BoB follow a distinct pattern, with hypoxic events most likely to occur between July and October, which coincides with the peak discharge of those river systems (Figure 10a). However, further offshore, hypoxic events are more likely to occur in February/March in the BoB and July/August in the Arabian Sea, consistent with summer wind‐driven upwelling and wave‐driven coastal Kelvin wave propagation around the rim of the northern Indian Ocean (Figure 10a). Interannual variations modulate this seasonal pattern. Coastal hypoxia is most likely during positive IOD phases in the BoB, during negative IOD phases in the EAS (especially between 13° N and 18° N), while other regions show a much weaker influence of IOD phases (Figure 10b).

## 4 Discussion and Conclusion

**FIGURE 11:**
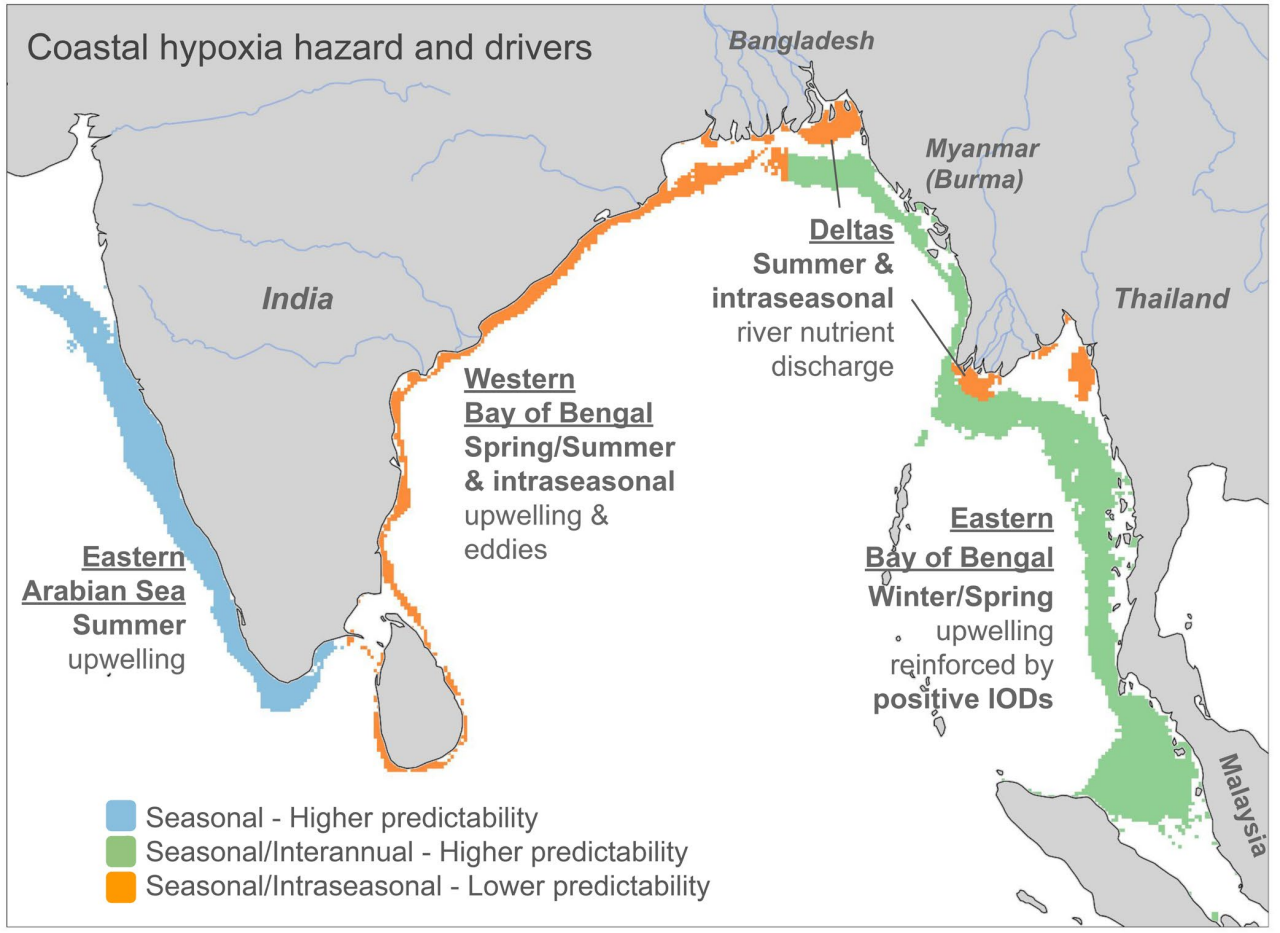
Basin‐scale mapping of coastal hypoxia timing and drivers in the eastern Arabian Sea, the western Bay of Bengal, the eastern Bay of Bengal and the major river deltas of the Bay of Bengal (Ganges–Brahmaputra and Irrawaddy–Sittang). Only coastal regions vulnerable to coastal hypoxia events are shown (i.e., occurrence likelihood between 0.01% and 99%). Colors indicate the dominant timescales of hypoxia and associated predictability: Blue marks areas with predominantly seasonal hypoxia and higher predictability; green indicates regions with both seasonal and interannual hypoxia, also with higher predictability; orange highlights regions influenced by both seasonal and intraseasonal variability, where predictability is lower. Map lines delineate study areas and do not necessarily depict accepted national boundaries.

In this study, we integrate *in*‐*situ* observations with a high‐resolution (1/12 degree resolution) biophysical model of the Indian Ocean to identify regions vulnerable to coastal hypoxia and to assess the spatiotemporal variability of coastal oxygen dynamics and its drivers. Our findings reveal a strong contrast between the eastern Arabian Sea, where hypoxia is controlled by seasonal upwelling; the western Bay of Bengal, where hypoxic events are often episodic and associated with intraseasonal variations in ocean circulation (currents, eddies and coastal Kelvin waves); the eastern Bay of Bengal, where hypoxia is strongly constrained by the interannual variability of the Indian Ocean Dipole; and major river deltas, where hypoxia is strongly tied to seasonal and intraseasonal variations in river discharge and biological production, as shown in Figure 11. Yet, we find that the hazard of hypoxia is often locked on the seasonal cycle, whether it is occurring on seasonal, intraseasonal, or interannual timescales. For instance, intraseasonal hypoxic events in the Ganges–Brahmaputra river delta systematically occur in summer, events along the northwestern Bay of Bengal occur in spring, and the interannual hypoxic events in the southeastern Bay of Bengal generally occur in spring of positive Indian Ocean Dipole years.

Physical dynamics largely control oxygen variations on continental shelves. The propagation of coastal Kelvin waves along the rim of the northern Indian Ocean controls the occurrence of hypoxia on seasonal and interannual time‐scales. This is revealed by the month of highest hypoxia hazard, which shifts anticlockwise around the northern Indian Ocean from January/February in the southeastern Bay of Bengal to March/April in the northwestern Bay of Bengal, and to July/August in the eastern Arabian Sea (Figure 10a). This shift is consistent with the timing of the coastal Kelvin wave propagation around the basin (Vinayachandran et al. 2021; Suresh et al. 2016; Nienhaus et al. 2012) and the associated upwelling of low oxygen waters onto the shelf identified from oxygen *in*‐*situ* observations (Pearson et al. 2022). On interannual time‐scales, our model shows that positive Indian Ocean Dipole phases intensify low‐oxygen conditions in the Bay of Bengal, contrasting with the eastern Arabian Sea where negative Indian Ocean Dipole phases have a more pronounced impact (Figure 10b). This pattern is consistent with the modulation of coastal Kelvin wave upwelling associated with the Indian Ocean Dipole (Rao et al. 2010; Suresh et al. 2016, 2018) and supports the work of Pearson et al. (2022) based on observational data and Vallivattathillam et al. (2017) based on modeling approaches that established a link between hypoxia occurrence and Indian Ocean Dipole phases. We find that the Indian Ocean Dipole has a stronger role in the eastern Bay of Bengal, where hypoxia occurs almost exclusively during positive Indian Ocean Dipole phases, consistent with the expectation that the interannual variability in upwelling motion is stronger in this region (Suresh et al. 2018).

Physical dynamics also control the occurrence of intraseasonal hypoxia events in the western Bay of Bengal. Their timing aligns with the strongest intraseasonal variations of the East India Coastal Current observed during spring (February–April), as reported by Mukherjee et al. (2014) using data from current profilers in the western Bay of Bengal region. Previous studies have also revealed that, in the Bay of Bengal, intraseasonal cyclonic and anticyclonic eddies can be predominantly influenced by intraseasonal wind anomalies modulated by the Madden Julian Oscillation in the equatorial Indian Ocean (Zhang et al. 2023; Webber et al. 2010). These wind anomalies trigger the generation of upwelling and downwelling Kelvin waves that traverse along the equator and subsequently propagate northward along the coast of Bay of Bengal. Further, we show that oxygen levels in this region are modulated by cyclonic mesoscale eddies, characterized by a shallower oxycline. These eddies are generated in the eastern Bay of Bengal and travel westward, reaching the western Bay of Bengal coastal waters (Cheng et al. 2017; Greaser et al. 2020; Nuncio and Kumar 2012).

Biological processes play a central role in hypoxia formation in river‐influenced regions of the northern Bay of Bengal, particularly during the summer and fall peak discharge seasons. In these areas, our model shows that nutrient‐rich river runoff leads to elevated primary production in surface waters, followed by intense subsurface oxygen consumption that drives oxygen levels toward near‐anoxic conditions. This biologically driven hypoxia intensifies from July to October, coinciding with peak runoff and stratification, and is reinforced by intraseasonal pulses in both oxygen consumption and lateral transport of low‐oxygen waters. These findings underscore the critical role of biological oxygen demand in shaping seasonal and intraseasonal hypoxia patterns in delta regions. While our model includes seasonal variability in river discharge, it assumes constant annual mean nutrient concentrations. This simplification likely biases the magnitude and variability of biological oxygen consumption, especially in the northern Bay of Bengal, where nutrient delivery is closely tied to river runoff. As a result, the model may underestimate the intensity and/or frequency of short‐term hypoxia events in deltaic regions (Pedde et al. 2017; Sarma 2022). Additionally, biological processes also contribute to oxygen dynamics on interannual timescales, with a clear link to Indian Ocean Dipole phases that drive basin‐scale biogeochemical signatures. Our results support and extend previous observational and modeling studies linking Indian Ocean Dipole phases to variability in primary productivity and coastal hypoxia. Consistent with satellite chlorophyll records (Wiggert et al. 2009; Currie et al. 2013), our model simulates enhanced productivity and more intense hypoxia in the eastern Bay of Bengal during positive Indian Ocean Dipole phases. Notably, our simulations reveal that hypoxia in the eastern Bay of Bengal occurs almost exclusively during positive Indian Ocean Dipole years, demonstrating a stronger link than previously captured.

Our basin‐scale mapping of hypoxia highlights both the challenges and opportunities in predicting and mitigating its effects across different regions (Figure 11). Coastal regions where hypoxia can be anticipated, such as the eastern Arabian Sea, where it occurs at the same time every year, or the eastern Bay of Bengal, where it occurs during a specific month and Indian Ocean Dipole phase, provide opportunities to prevent or mitigate the adverse impacts on coastal ecosystems and the services they provide. For instance, seasonal forecasting systems have been used to inform fisheries management and mitigate adverse impacts on marine species (e.g., J‐SCOPE in the Pacific Northwest: Norton et al. 2023). Forecasts provide actionable information that allows managers to plan ahead, adjust fishing schedules, and, in some cases, close fisheries temporarily to prevent over exploitation or damage to the ecosystem during hypoxic events. Examples include the 2018 early closure of the Dungeness crab fishery by the Quinault Indian Nation due to predicted hypoxic conditions (Stoltz et al. 2021; Siedlecki et al. 2023), and the Mississippi River/Gulf of Mexico Hypoxia Task Force, which implemented decision support tools that integrate ecosystem models with stakeholder inputs to guide management actions and mitigate hypoxia's impact on fisheries (Shaffer et al. 2023). In addition to fisheries management, the ability to anticipate hypoxic events would facilitate the use of mitigation techniques, such as artificial oxygenation to offset hypoxia and relieve marine ecosystems deoxygenation stress (e.g., Handmann and Wallace 2024). In contrast, regions where intraseasonal variability can reinforce seasonal hypoxia, such as the western Bay of Bengal or river deltas, present a significant challenge for prevention and mitigation. The timing and magnitude of these episodes are difficult to anticipate due to their short timescales and complex interactions with physical and biological processes. Enhanced monitoring and early warning systems relying on remote sensing and real‐time data could be key to reducing ecological and socioeconomic damage in these areas.

## Author Contributions

**Fan Yang:** conceptualization, data curation, formal analysis, investigation, methodology, resources, software, validation, visualization, writing – original draft, writing – review and editing. **Laure Resplandy:** conceptualization, formal analysis, funding acquisition, investigation, methodology, project administration, resources, supervision, writing – original draft, writing – review and editing. **Yangyang Zhao:** software, writing – review and editing. **Sam Ditkovsky:** software, writing – review and editing.

## Conflicts of Interest

The authors declare no conflicts of interest.

## Supporting information


**Data S1:** gcb70378‐sup‐0001‐DataS1.pdf.

## Data Availability

The data that support the findings of this study are openly available at https://doi.org/10.5281/zenodo.14199666.
