# Supplementary material for: Coastal Hypoxia in the Indian Ocean: Unraveling Drivers of Spatio‐Temporal Variability
Source: Glob Chang Biol. 2025 Aug 6;31(8):e70378. doi: 10.1111/gcb.70378 (PMC12326294; doi:10.1111/gcb.70378)
Supplement: Supplementary file 1 — Data S1: gcb70378‐sup‐0001‐DataS1.pdf. [file GCB-31-e70378-s001.pdf]

Supporting Information for "Coastal hypoxia in the Indian Ocean: unraveling drivers of spatio-temporal variability"

Fan Yang[1], **Laure Resplandy**[1], **Yangyang Zhao**[1], **Sam Ditkovsky**[2]

[1]Department of Geosciences and High Meadows Environmental Institute, Princeton University, Princeton, NJ, USA
[2]Program in Atmospheric and Oceanic Sciences, Princeton University, Princeton, NJ, USA

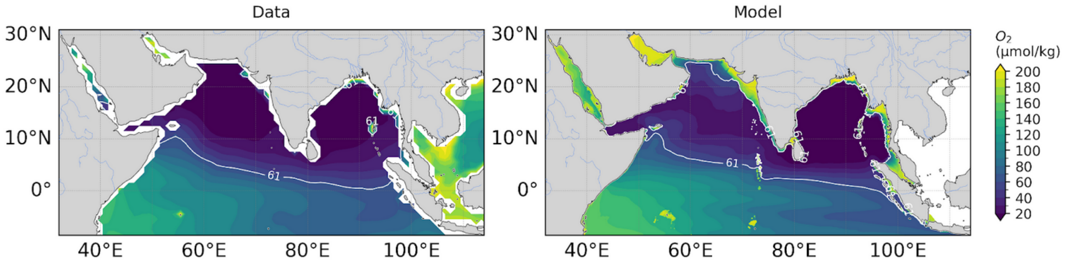

FIGURE S1 (a) Observation-based minimum oxygen concentrations (μmol/kg) in the upper 200 m from Bianchi et al. (2012). (b) Modeled minimum oxygen concentrations in the upper 200 m from MOM6-COBALT-IND12, averaged over 1980–2020. The 61 μmol/kg contours (white line) denote the hypoxia threshold.

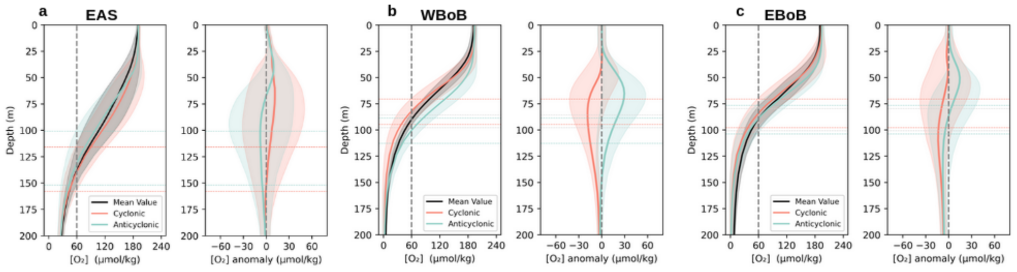

FIGURE S2 Oxygen concentration and anomalies profiles as a function of depth beneath the centroids of cyclonic eddies (red) and anticyclonic eddies (cyan) in the upper 200 m in the (a) EAS, (b) WBOB, and (d) EBoB regions. Horizontal dashed lines indicate the depth at which oxygen levels fall below hypoxic conditions.

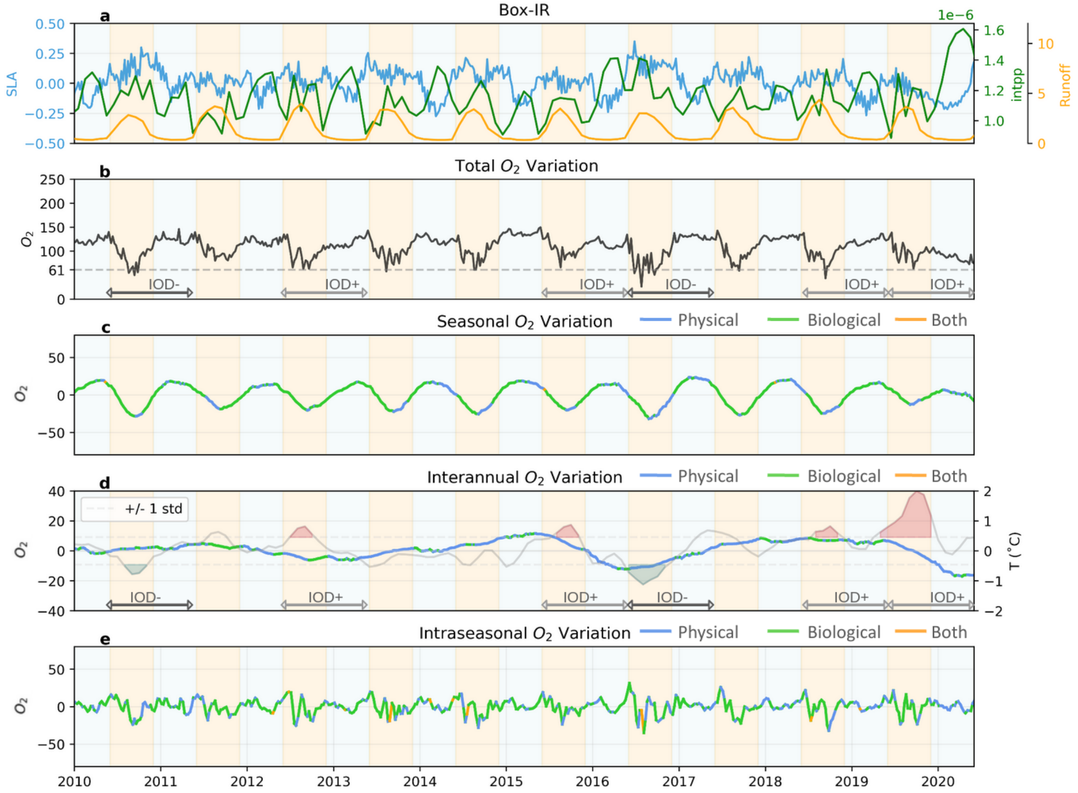

FIGURE S3 Oxygen dynamics in the Bay of Bengal river delta regions Box-IR from year 2010 to 2020. (a) sea level anomalies (SLA, m), integrated primary production in the upper 100 m (intPP, mol m^{-2} s^{-1}) and river runoff (10^4 m^3 s^{-1}); (b-e) Decomposition of oxygen variability (averaged between 5-20 m, μmol/kg) across total, seasonal, interannual, and intraseasonal timescales. The main drivers of oxygen variability on seasonal, interannual, and intraseasonal timescales are indicated by color: blue where physical processes dominate, green where biological processes dominate, and orange where both physical and biological processes actively contribute to the oxygen changes. The yellow shading indicates the summer/fall period (June to November). In panel (d), green and red shadings represent negative and positive IOD phases, respectively.

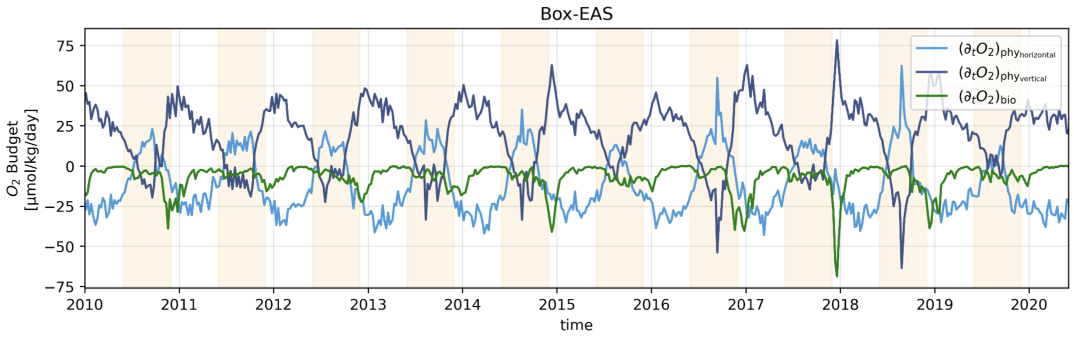

FIGURE S4 Oxygen budget terms in Box-EAS from year 2010 to 2020, averaged over the 40–200 m depth range. The light blue line $(\partial_t O_2)$ phy$_{\text{horizontal}}$ represents horizontal physical transport. The dark blue line $(\partial_t O_2)$ phy$_{\text{vertical}}$ represents vertical physical transport. The green line $(\partial_t O_2)_{\text{bio}}$ represents all biological contributions. Negative values of the total biological oxygen tendency indicate that biological consumption exceeds production.

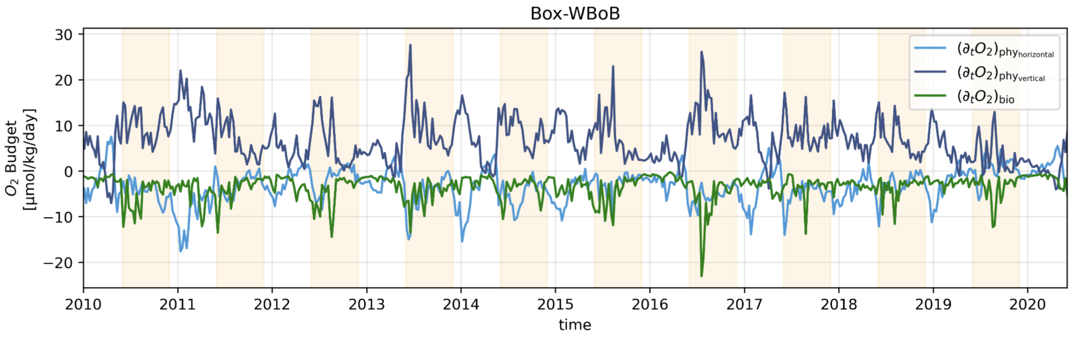

FIGURE S5 Same as Figure S4, but for the Box-WBoB region.

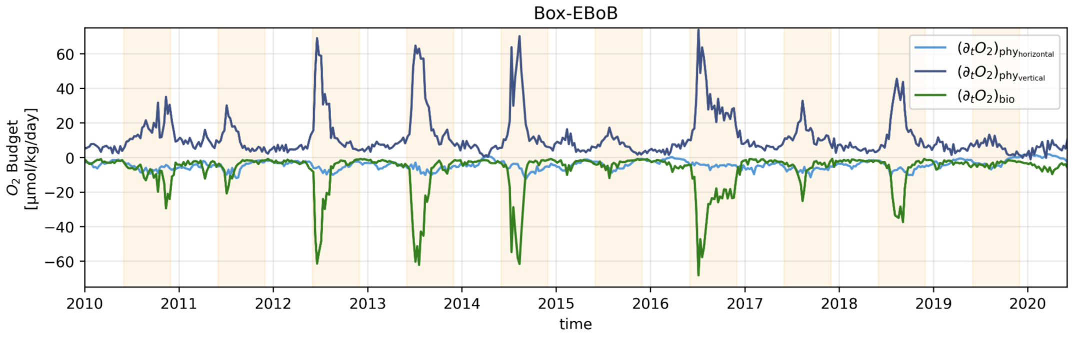

FIGURE S6 Same as Figure S4, but for the Box-EBoB region.

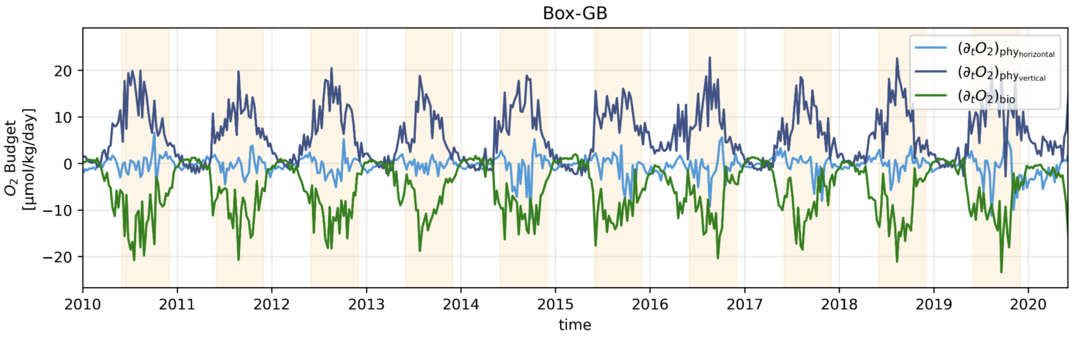

FIGURE S7 Same as Figure S4, but for the Box-GB region, averaged between 5-20 m.

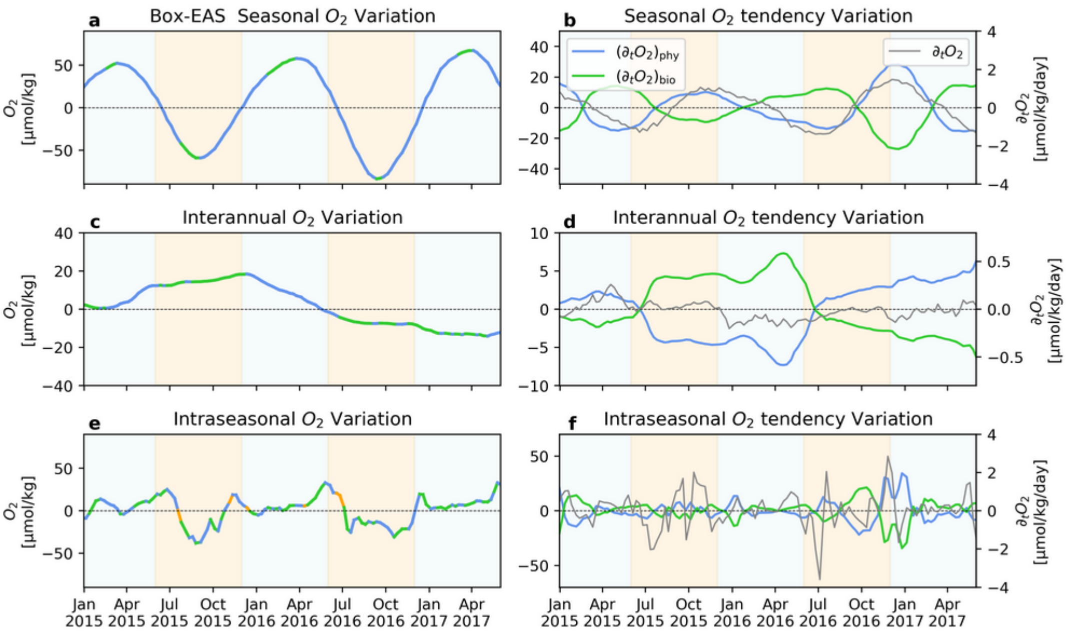

FIGURE S8 Decomposition of oxygen variability (left) and oxygen budget terms (right) in the Box-EAS region from January 2015 to June 2017. Results are shown for three timescales: seasonal (top row), interannual (middle row), and intraseasonal (bottom row). Left panels show modeled oxygen variability (averaged between 40–200 m, μmol/kg), with dominant drivers indicated by color: blue where physical processes dominate, green where biological processes dominate, and orange where both contribute. Right panels show corresponding budget terms, with physical and biological contributions on the left axis and total oxygen tendency (gray) on the right. Yellow shading highlights the summer/fall period (June to November).

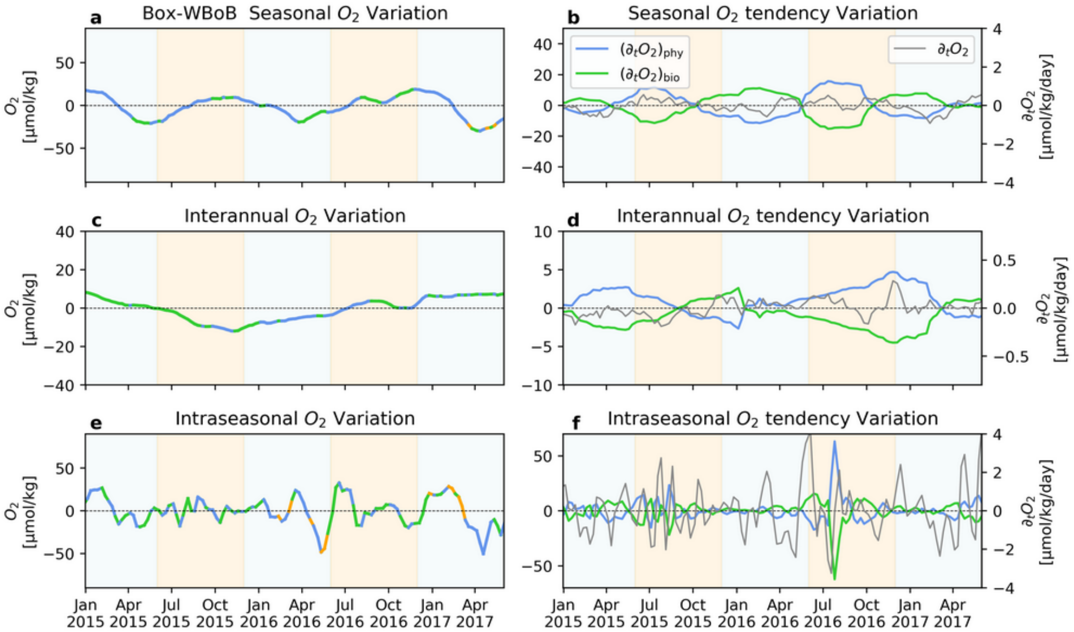

FIGURE S9 Same as Figure S8, but for the case study region Box-WBoB.

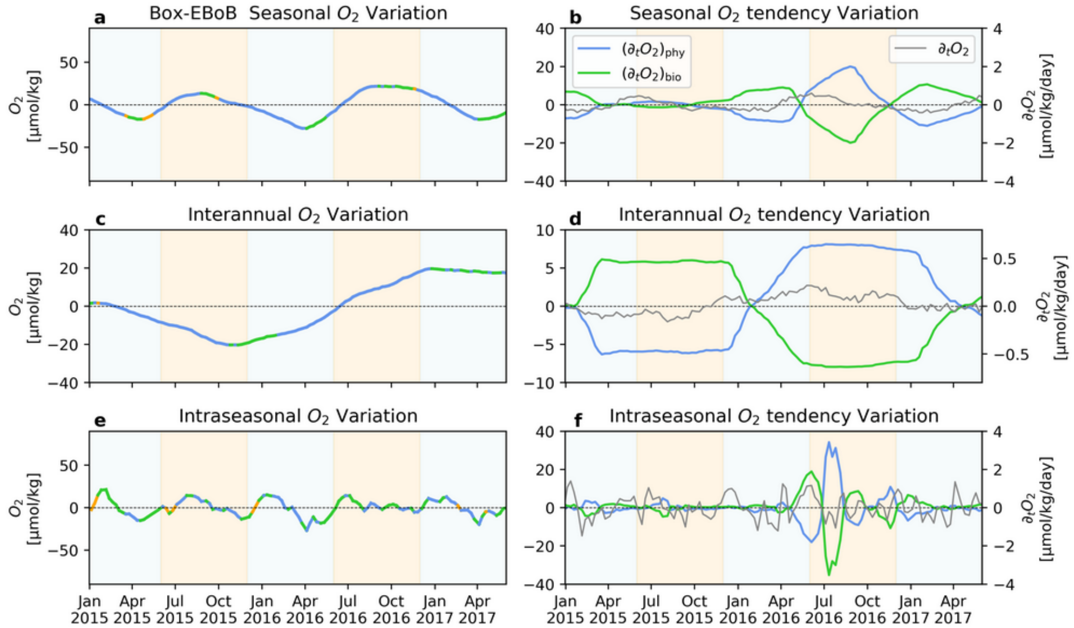

FIGURE S10 Same as Figure S8, but for the case study region Box-EBoB.

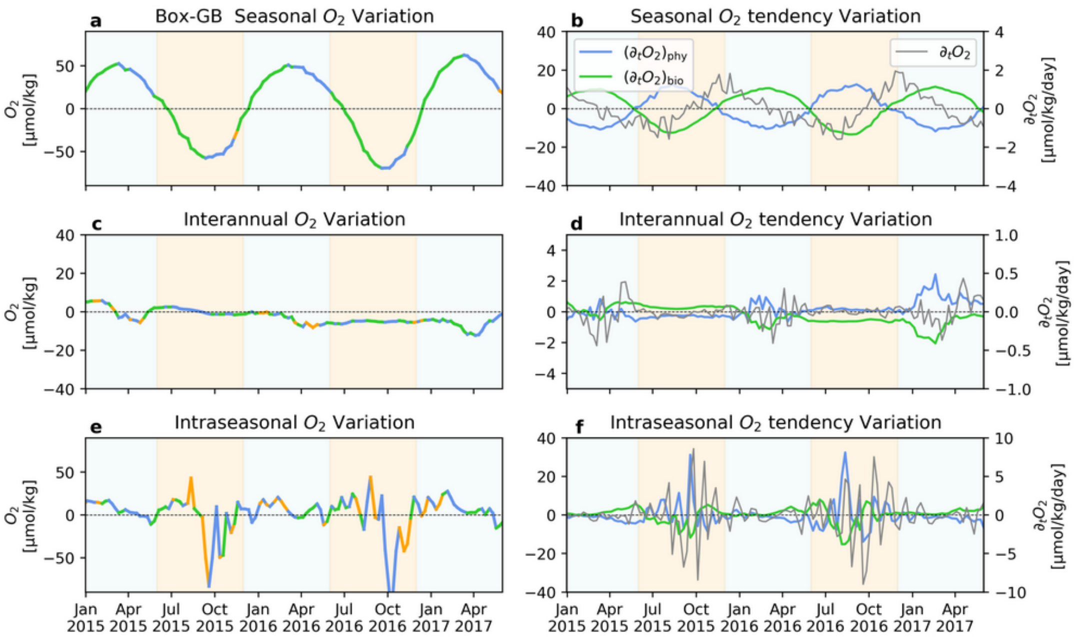

FIGURE S11 Same as Figure S8, but for the case study region Box-GB, with oxygen averaged between 5–20 m.

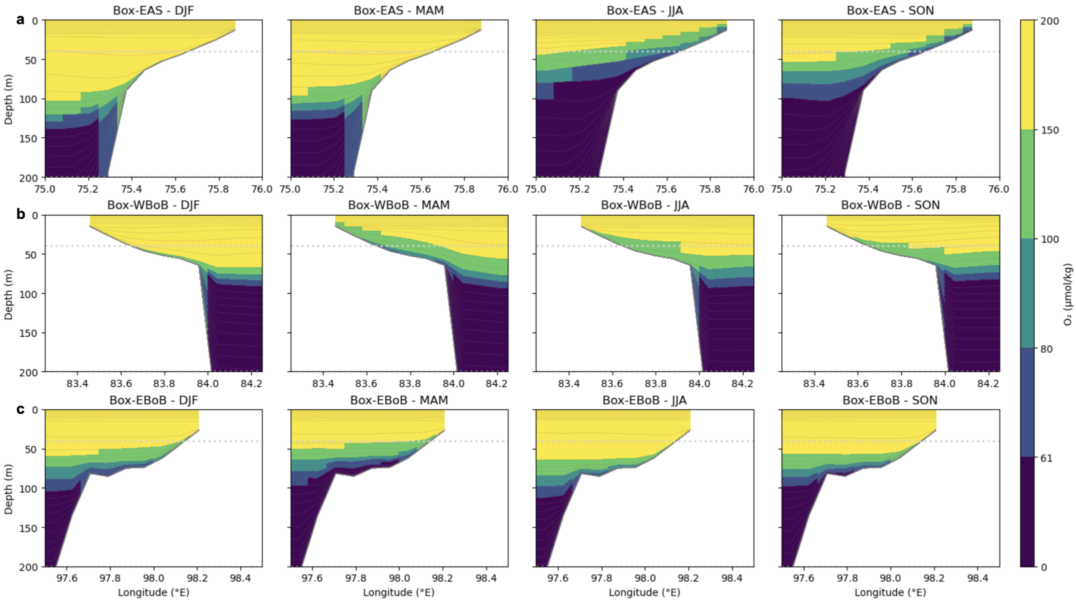

FIGURE S12 Zonal sections of oxygen (µmol/kg) for (a) Box-EAS (top row), (b) Box-WBoB (middle row), and (c) Box-EBoB (bottom row) during four seasons: winter (December to February), spring (March to May), summer (June to August), and fall (September to November). Each panel displays the seasonal mean oxygen concentration along longitude and depth. Grey dashed lines indicate the 40 m depth.
